# A De Giorgi conjecture on the regularity of minimizers of Cartesian area in 1D

**DOI:** 10.1007/s11565-026-00732-2

**Published:** 2026-09-08

**Authors:** Giovanni Bellettini, Shokhrukh Yu Kholmatov

**Affiliations:** 1 Dipartimento di Scienze Matematiche, Informatiche e Fisiche, via delle Scienze 206, 33100 Udine, Italy; 2 International Centre for Theoretical Physics (ICTP), Strada Costiera 11, 34151 Trieste, Italy; 3 University of Vienna, Oskar-Morgenstern-Platz 1, 1090 Vienna, Austria; 4 Samarkand State University, University boulevard 15, 140104 Samarkand, Uzbekistan

**Keywords:** Cartesian area, Anisotropic area functional, $L^p$-perturbations, *BV*-functions, De Giorgi conjecture, 49Q15, 49Q20, 26A06, 26A45

## Abstract

We prove a $C^{1,1}$-regularity result for minimizers of the functional

$$ \int _I \sqrt{1+|Du|^2} + \int _I |u-g|ds,\quad u\in BV(I), $$

provided $I\subset \mathbb {R}$ is a bounded open interval and $\Vert g\Vert _\infty$ is sufficiently small, thus partially establishing a De Giorgi conjecture in dimension one and codimension one. We also extend our result to a suitable anisotropic setting.

## Introduction

The non-parametric minimal surfaces, more generally, the prescribed mean curvature surfaces, have been extensively studied in the literature from the variational perspective (see e.g. [11, 14, 16–18] and the references therein). Given an open set $\Omega \subset \mathbb {R}^n$ and a sufficiently regular function $H:\Omega \rightarrow \mathbb {R},$ the underlying equation is rewritten as

1.1 $$\begin{aligned} \textrm{div}\frac{\nabla u}{\sqrt{1+|\nabla u|^2}} = H\quad \text {in } \Omega \end{aligned}$$

with a prescribed Dirichlet or Neumann boundary condition, and corresponds to the Euler-Lagrange equation of the functional

1.2 $$\begin{aligned} \int _\Omega \sqrt{1+|\nabla u|^2}dx + \int _\Omega Hu\,dx,\quad u\in C^1(\Omega ). \end{aligned}$$

It is well-known that under suitable assumptions on Ω and *H*,  the minimizers are in fact locally $C^{2+\alpha },$ and hence solve (1.1) in a classical sense.

In the context of functionals with linear growth, a related problem is the existence and regularity of minimizers of the (convex, but not strictly convex) functional

$$ \mathscr {F}(u):=\int _\Omega \sqrt{1+|\nabla u|^2}dx + \int _\Omega |u-g|\,dx,\quad u\in C^1(\Omega ), $$

where $g\in L^1(\Omega )$ is given, see [7]. In this case, the associated Euler-Lagrange equation becomes formally a differential inclusion of the form

1.3 $$\begin{aligned} \textrm{div}\frac{\nabla u}{\sqrt{1+|\nabla u|^2}} \in {\left\{ \begin{array}{ll} \{1\} &  \text {in } \{u>g\},\\ {[}-1,1] &  \text {in }\{u=g\}, \\ \{-1\} &  \text {in } \{u<g\}, \end{array}\right. } \end{aligned}$$

thus, in the sets $\{u>g\}$ and $\{u<g\},$ the subgraph of *u* has mean curvature equal to 1 and -1, respectively.

Unlike the minimizers of the functional in (1.2), the equation (1.3) may admit nonregular solutions, as observed in [7]. For instance, if n=1, Ω=(-1,1) and

$$ g(s) = {\left\{ \begin{array}{ll} 2 &  \text {if } s\in (0,1),\\ -2 &  \text {if } s\in (-1,0), \end{array}\right. } $$

one can readily check that the functions

1.4 $$\begin{aligned} u_{a,b}(s) = {\left\{ \begin{array}{ll} \sqrt{2s-s^2} + a &  \text {if } s\in (0,1),\\ \sqrt{-2s-s^2} + b &  \text {if } s\in (-1,0) \end{array}\right. } \end{aligned}$$

with $-1\le b\le a\le 1$ satisfy (1.3) and minimize $\mathscr {F},$ with $\mathscr {F}(u_{a,b})=4+\frac{\pi }{2}.$ However, any $u_{a,b}$ is not continuously differentiable at s=0, worse still, it has a jump if a>b.

To study regularity of minimizers of $\mathscr {F},$ in [7] De Giorgi posed the following conjecture, which seems nontrivial even when n=k=1.

### Conjecture 1.1

For any n,k≥1, there exists $\sigma :=\sigma (n,k)>0$ such that for any open ball $B\subset \mathbb {R}^n$ and $g\in L^\infty (B;\mathbb {R}^k)$ with $\Vert g\Vert _\infty \le \sigma$ the following minimum is achieved:

1.5 $$\begin{aligned} \min \Big \{\int _B \sqrt{1+\sum |M_i(\nabla u)|^2} dx + \int _B |u-g|dx:\,\,u\in C^1(B;\mathbb {R}^k)\Big \}, \end{aligned}$$

where the sum is taken over all minors $M_i(\nabla u)$ of the Jacobian matrix ∇u of *u*.

A variation of this conjecture for n=1 and k≥1 has been recently addressed in [5]: using the Sobolev regularity theory for the minimizers of an Ambrosio-Tortorelli-type functional [2], the authors have shown the existence of $\sigma :=\sigma (k,|I|^{-1/2})>0,$ such that for any $g\in L^\infty (I;\mathbb {R}^k)$ with $\Vert g\Vert _\infty \le \sigma ,$ the minimum problem

1.6 $$\begin{aligned} \min \Big \{\int _I \sqrt{1+|u'|^2}\,ds+ \int _I (u-g)^2\,ds:\,\, u\in C^1(I;\mathbb {R}^k)\Big \} \end{aligned}$$

admits a unique solution, where *I* is a bounded interval. This result does not solve Conjecture 1.1, due to the exponent 2 in the second integral of the functional and to the dependence of σ on the length |*I*| of the interval *I*. To prove the existence of solutions, they observe that if $u\in W^{1,\infty }(I;\mathbb {R}^k)$ minimizes the Γ-limit *F* of a suitable sequence of approximating functionals, then it also minimizes the functional in (1.6), which turns out to be Sobolev regular provided that $\Vert g\Vert _\infty$ is small enough depending only on |*I*|. Next, they show that *u* is in fact a solution to the corresponding Euler-Lagrange equation with suitable boundary conditions, which yield the continuity of the derivative (here the quadratic term $(u-g)^2$ is important in the analysis of the Euler-Lagrange equation).

In the present paper we consider n=k=1 and generalize the functional in (1.5) to the anisotropic case with $L^p$-fidelity terms. Given an anisotropy (i.e., a norm) φ on $\mathbb {R}^2,$ $p\in [1,+\infty ),$ a bounded open interval $I\subset \mathbb {R}$ and $g\in L^\infty (I),$ we consider the functional

1.7 $$\begin{aligned} \mathscr {G}(u) = \int _I\varphi ^o(-Du,1) + \int _I|u-g|^pds,\quad u\in L^1(I), \end{aligned}$$

where $\varphi ^o$ is the dual of φ and

$$ \int _I \varphi ^o(-Du,1):=\sup \Big \{\int _I (uh_1'+h_2)ds:\,\, (h_1,h_2)\in C_c^1(I;\mathbb {R}^2),\,\, \Vert \varphi (h_1,h_2)\Vert _\infty \le 1\Big \} $$

is the φ-total variation of $(-Du,\mathcal {L}^1)$ when u∈BV(I). The main result of this paper reads as follows (see also Theorem 5.4).

### Theorem 1.2

Let φ be an anisotropy on $\mathbb {R}^2$ such that the unit ball $W^\varphi :=\{\varphi \le 1\}$ is symmetric with respect to the coordinate axes and does not have vertical facets. Let $I\subset \mathbb {R}$ be a bounded open interval. Then there exists $\sigma :=\sigma (\varphi ,p,|I|)>0$ such that for any $g\in L^\infty (I)$ with $\Vert g\Vert _\infty < \sigma$ every minimizer of $\mathscr {G}$ is Lipschitz continuous in *I*. Additionally, if φ is $C^2$ away from the origin and elliptic (see Definition 2.1), then minimizers are $C^{1,1}$ in *I*.

In the Euclidean case $\varphi =|\cdot |,$ Theorem 1.2 provides a positive solution to Conjecture 1.1 for n=k=1, except that our σ depends on |*I*| (as in [5]); at the same time we gain an extra regularity of minimizers.

To prove Theorem 1.2, we begin by observing that if *g* is bounded, then every minimizer *u* of $\mathscr {G}$ is also bounded, with $\Vert u\Vert _\infty \le \Vert g\Vert _\infty$ (see Lemma 5.1). If, additionally, φ is even in each coordinate (equivalently, $W^\varphi$ is symmetric with respect to the coordinate axes), then the subgraph and the epigraph of *u* are $(\gamma ,\Lambda )$-local minimizers of the φ-perimeter, in the sense of Definition 4.5 below, with suitable constants $\gamma ,\Lambda$ (Proposition 5.3). Next in Proposition 4.3, we prove that if *u* satisfies an appropriate $L^\infty$-bound depending only on Λ and |*I*|,  a tangent φ-ball condition holds at each point *x* on the graph of *u* for all radii *r* up to $\frac{\alpha _0}{\Lambda }>0$ for a constant $\alpha _0>0$ depending only on φ. The uniformity of the radii of these tangent balls allows us to estimate the deviation of the generalized normals to the reduced boundary of the subgraph of *u* in $I\times \mathbb {R}$ from the vertical direction (see (4.14)) which is away from 0 provided that $\Vert u\Vert _\infty$ is small enough depending only on φ, Λ and |*I*|. In particular, this observation and [19, Lemma 3.10] imply that *u* is Lipschitz continuous in *I* (Corollary 4.4). Finally, an explicit choice of σ is made, using the previous $L^\infty$-bounds for *u* (Theorem 5.4). When φ is $C^2$ and elliptic, the tangent φ-ball condition becomes equivalent to the classical tangent ball condition, and hence *u* must be $C^{1,1}$ in *I*.

Note that the function $u_{a,b}$ in (1.4) shows that in case $\Vert g\Vert _\infty$ is large, the validity of a tangent ball condition may not suffice for the regularity of minimizers of $\mathscr {F}.$

When Λ=0 and $\gamma =+\infty ,$ $(\gamma ,\Lambda )$-local minimizers coincide with classical local minimizers. In this case, assuming φ is symmetric with respect to the coordinate axes, we can characterize all possible Cartesian local minimizers of the φ-perimeter (see Theorem 3.1).

The paper is organized as follows. In Sect. 2 we introduce some preliminaries on anisotropies, Λ-local minimizers, and φ-ball condition for Cartesian Λ-local minimizers. In Sect. 3 we provide a characterization of local minimizers. Some regularity properties of Λ-local minimizers and their further generalizations are studied in Sect. 4. Finally, we prove Theorem 1.2 in Sect. 5.

We dedicate this article to Umberto Massari, whose papers, as well as his book on minimal surfaces written in collaboration with M. Miranda, have been a lasting source of mathematical inspiration.

## Some preliminaries

In what follows, by $\mathcal {L}^m$ (typically for m=1,2) and $\mathcal {H}^t$ we denote the Lebesgue measure in $\mathbb {R}^m$ and the *t*-dimensional Hausdorff measure in $\mathbb {R}^2.$ Depending on the context, we use |·| to denote the Euclidean norm of a vector in $\mathbb {R}^2,$ the length of a bounded interval on $\mathbb {R}$ and the measure of a (Lebesgue) measurable set in $\mathbb {R}^m$ for m=1,2. The scalar product in $\mathbb {R}^2$ is indicated by $\left\langle \cdot ,\, \cdot \right\rangle .$ The symmetric difference of sets *A* and *B* is denoted AΔB. The symbol $B_r(x)$ stands for the Euclidean ball in $\mathbb {R}^2$ centered at *x* and of radius r>0. The topological closure, interior and boundary of $E\subset \mathbb {R}^2$ will be denoted by $\overline{E},$ $\mathring{E}$ and ∂E, respectively. Given an open interval $I\subset \mathbb {R},$ we write $\mathscr {O}(I)$ and $\mathscr {O}_b(I)$ to denote the collection of all open and all bounded open subsets of *I*,  respectively.

### Anisotropies

Let $\varphi :\mathbb {R}^2\rightarrow [0,+\infty )$ be an anisotropy, i.e., a positively one-homogeneous even convex function with

2.1 $$\begin{aligned} c\le \varphi \le \frac{1}{c} \quad \text {on the unit circle } \mathbb {S}^1 \end{aligned}$$

for some c∈(0,1]. We denote by $\varphi ^o$ the dual of φ, defined as

$$ \varphi ^o(\xi ) = \max _{\varphi (\eta )=1}\,\left\langle \xi ,\, \eta \right\rangle , $$

which is also an anisotropy on $\mathbb {R}^2.$ We say that φ is a $C^k$-anisotropy for some k≥1 provided that $\varphi \in C_{\textrm{loc}}^k(\mathbb {R}^2\setminus \{0\}).$

The unit φ-ball $W^\varphi :=\{\varphi \le 1\}$ is sometimes called the Wulff shape of φ. We also introduce the φ-ball of radius *r* centered at *x* as $W_r^\varphi (x):=\{\varphi (\cdot -x)\le r\};$ clearly $\mathring{W}_r^\varphi (x)=\{\varphi (\cdot -x)<r\}.$ Given $\eta \in \partial W^\varphi ,$ we call any vector $\nu \in \partial \varphi ^o(\eta )$ a *normal* to $W^\varphi$ at η, where ∂ is the subdifferential. Notice that if $W^\varphi$ is not regular at η, for instance, it has a corner, its set of normals at η forms a nonempty closed convex cone.

We write $\textrm{dist}_\varphi (x,S):=\inf \{\varphi (x-y):\,\,y\in S\}$ to denote the φ-distance function from a nonempty set *S*.

#### Definition 2.1

(Elliptic anisotropy) An anisotropy φ on $\mathbb {R}^2$ is *elliptic* provided that there exists λ>0 such that $\phi (\cdot ) - \lambda |\cdot |$ is also an anisotropy on $\mathbb {R}^2.$

For instance, any anisotropy induced by some positive definite quadratic form, is elliptic. The following proposition can be found in [13, Appendix A] and provides a characterization of elliptic $C^2$-anisotropies.

#### Proposition 2.2

For any $C^2$-anisotropy the following assertions are equivalent:

- φ is elliptic;
- $\varphi ^o$ is $C^2$ and elliptic;
- there exists $\bar{r}\in (0,1)$ such that for any $z\in \partial W^\varphi$ there exist $x_z,y_z\in \mathbb {R}^2$ such that
  $$ B_{\bar{r}}(x_z)\subset W^\varphi \subset \overline{B_{1/\bar{r}}(y_z)} \qquad \text {and}\qquad (\partial B_{\bar{r}}(x_z))\cap \partial W^\varphi =(\partial B_{1/\bar{r}}(y_z))\cap \partial W^\varphi = \{z\}. $$

Another interesting class of anisotropies is introduced in [4, Sect. 4]:

#### Definition 2.3

We say an anisotropy φ is *partially monotone* if

$$ (x_1,y_1),(x_2,y_2)\in \mathbb {R}^2,\quad |x_1|\le |x_2|,\,\,\,|y_1|\le |y_2|\qquad \Longrightarrow \quad \varphi (x_1,y_1)\le \varphi (x_2,y_2). $$

According to [4, Appendix A] the following statements are equivalent:

- φ is partially monotone;
- $\varphi ^o$ is partially monotone;
- $$\varphi (x_1,x_2)=\varphi (|x_1|,|x_2|)$$ for all $$x_1,x_2\in \mathbb {R}.$$

Thus, φ is partially monotone if and only if it is even in each coordinate separately. Equivalently, φ is partially monotone if and only if its Wulff shape $W^\varphi$ is symmetric with respect to the coordinate axes.

### Anisotropic total variation and perimeter

Let φ be an anisotropy on $\mathbb {R}^2$ and $I\subseteq \mathbb {R}$ be an open interval. Recall that a function $u:I\rightarrow \mathbb {R}$ has locally bounded variation in *I*,  and we write $u\in BV_{\textrm{loc}}(I),$ if its distributional derivative *Du* is a Radon measure in *I*. If, additionally, $u\in L^1(I)$ and *Du* is a bounded Radon measure in *I*, then *u* is called a function of bounded variation and is denoted by u∈BV(I).

Given $u\in BV_{\textrm{loc}}(I),$ the anisotropic area of the graph of $u\in BV_{\textrm{loc}}(I)$ is defined by the φ-total variation of the Radon measure $(-Du,\mathcal {L}^1)$ in an open set J⋐I as

$$\begin{aligned} \mathscr {A}_\varphi (u,J)  &   := \int _J \varphi ^o(-Du,1)\\  &   := \sup \Big \{\int _{J} (uh_1' + h_2)ds:\,\, (h_1,h_2)\in C_c^1(J;\mathbb {R}^2),\,\,\Vert \varphi (h_1,h_2)\Vert _\infty \le 1\}. \end{aligned}$$

When $u\in W_{\textrm{loc}}^{1,1}(I),$ the Radon-Nikodym theorem implies

$$ \mathscr {A}_\varphi (u,J) = \int _J \varphi ^o(-u',1)ds, $$

and hence, in the case of the Euclidean anisotropy,

$$ \mathscr {A}_{|\cdot |}(u,J) = \int _J \sqrt{1+{u'}^2}\,ds. $$

Note that for $u\in BV_{\textrm{loc}}(I)$ we have [6, p. 390]

2.2 $$\begin{aligned} \mathscr {A}_\varphi (u,I) =&\int _I \varphi ^o(-u',1)ds+ \varphi ^o(D^su,0)(I) \nonumber \\ =&\int _I \varphi ^o(-u',1)ds+ \sum _{x\in J_u} \varphi ^o(\textbf{e}_1)|u^+(x)-u^-(x)| + \varphi ^o(D^cu,0)(I), \end{aligned}$$

where $D^su$ and $D^cu$ are the singular part of *Du* with respect to $\mathcal {L}^1$ and the Cantor part respectively, $J_u$ is the jump set of *u*,  $u^\pm (x)$ are the right and left traces of *u* at *x* and

$$ \varphi ^o(\mu ,0)(I) = \sup \Big \{\int _I \eta \,d\mu :\,\, \eta \in C_c(I),\,\,\Vert \varphi (\eta ,0)\Vert _\infty \le 1\Big \} $$

is the partial φ-total variation of a Radon measure μ in *I*.

A measurable set $E\subset \mathbb {R}^2$ is called of locally finite perimeter in an open set $\Omega \subseteq \mathbb {R}^2,$ and denoted as $E\in BV_{\textrm{loc}}(\Omega ;\{0,1\}),$ provided that the distributional derivative $D\chi _E$ of its characteristic function $\chi _E$ is a Radon measure in Ω. If, additionally, $D\chi _E$ is a bounded Radon measure in Ω, then *E* has finite perimeter. We denote by $\partial ^*E$ and $\nu _E$ the reduced boundary and the generalized outer unit normal of *E*,  respectively. If $\chi _E\in BV(\mathbb {R}^2),$ we write $E\in BV(\mathbb {R}^2;\{0,1\}).$ We refer for instance to [1, 11, 15, 17] for more information on *BV*-functions and sets of finite perimeter.

We define the φ-perimeter of *E* in the open set $\Omega \subseteq \mathbb {R}^2$ as

$$ P_\varphi (E,\Omega ) = \int _{\Omega \cap \partial ^*E} \varphi ^o(\nu _E)d\mathcal {H}^1. $$

We also set $P(E,\Omega ):= P_{|\cdot |}(E,\Omega )$ and $P_\varphi (E):=P_\varphi (E,\mathbb {R}^2)$

For a function $u:I\rightarrow \mathbb {R}$ we write

$$ \textrm{sg}(u):=\{(s,t)\in I\times \mathbb {R}:\,\, \,u(s)>t\}\subset \mathbb {R}^2 $$

to denote the (strict) subgraph of *u* (sometimes called hypograph). There is a natural connection between the anisotropic area of the graph and the anisotropic perimeter of the subgraph.

#### Lemma 2.4

[6] $u\in BV_{\textrm{loc}}(I)$ if and only if $\textrm{sg}(u)$ has locally finite perimeter in $I\times \mathbb {R}.$ Moreover, in either case, for any J⋐I,

$$ \mathscr {A}_\varphi (u,J) = P_\varphi (\textrm{sg}(u),J\times \mathbb {R}). $$

### Local minimizers

In this section we recall the notion of Λ-local minimizer.

#### Definition 2.5

Let φ be an anisotropy on $\mathbb {R}^2$ and $\Lambda \ge 0.$

- We call a function $u\in BV_{\textrm{loc}}(I)$ a Λ-local minimizer of $\mathscr {A}_\varphi$ in *I* if
  $$ \mathscr {A}_\varphi (u,J) \le \mathscr {A}_\varphi (u+\psi ,J) + \Lambda \int _J|\psi |ds$$
  for any $J\in \mathscr {O}_b(I)$ and $\psi \in C_c^1(J).$
- For an open set $\Omega \subseteq \mathbb {R}^2$ and $\Lambda \ge 0,$ we call a set $E\in BV_{\textrm{loc}}(\Omega ;\{0,1\})$ a Λ-local minimizer of $P_\varphi$ in Ω if
  $$ P_\varphi (E,\Omega ') \le P_\varphi (F,\Omega ') + \Lambda |E\Delta F| $$
  for any open $\Omega '\Subset \Omega$ and $F\in BV_{\textrm{loc}}(\Omega ;\{0,1\})$ with $E\Delta F\Subset \Omega '.$

When Λ=0, following the literature, we shortly call *u* (resp. *E*) a local minimizer.

By approximation, one can show that $u\in BV_{\textrm{loc}}(I)$ is a Λ-local minimizer of $\mathscr {A}_\varphi$ in *I* if and only if

$$ \mathscr {A}_\varphi (u,J) \le \mathscr {A}_\varphi (v,J) + \Lambda \int _J|u-v|ds$$

for any $J\in \mathscr {O}_b(I)$ and $v\in BV_{\textrm{loc}}(I)$ with $\mathrm {supp\,}(u-v)\Subset J.$

#### Remark 2.6

If *I* is bounded and $u\in L^\infty (I)\cap BV_{\textrm{loc}}(I)$ is a Λ-local minimizer of $\mathscr {A}_\varphi$ in *I*,  then u∈BV(I). Indeed, for any open interval J⋐I consider the test function $v=u\chi _{I\setminus J}.$ Then

$$ \mathscr {A}_\varphi (u,J) \le \varphi (\textbf{e}_2)|J| + 4\Vert u\Vert _\infty + 2\Lambda \Vert u\Vert _\infty |J|. $$

Now letting J↗I we find $\mathscr {A}_\varphi (u,I)<+\infty$ and hence u∈BV(I). In particular, the traces of *u* on ∂I are well-defined.

These two notions are linked as follows.

#### Proposition 2.7

Let $$u\in BV_{\textrm{loc}}(I).$$

- If the subgraph $\textrm{sg}(u)$ is a Λ-local minimizer of $P_\varphi$ in $I\times \mathbb {R},$ then *u* is a Λ-local minimizer of $\mathscr {A}_\varphi$ in *I*.
- If φ is partially monotone and *u* is a Λ-local minimizer of $\mathscr {A}_\varphi$ in *I*,  then $\textrm{sg}(u)$ is a Λ-local minimizer of $P_\varphi$ in $I\times \mathbb {R}.$

At the moment we do not have any explicit example showing the necessity of partial monotonicity in the second assertion of the proposition.

#### Proof

Let $\textrm{sg}(u)$ be a Λ-local minimizer of $P_\varphi$ in $I\times \mathbb {R},$ and fix $J\in \mathscr {O}_b(I)$ and $\psi \in C_c^1(J).$ Then $\textrm{sg}(u)\Delta \textrm{sg}(u+\psi ) \Subset J\times \mathbb {R}$ and hence, for any bounded open set $\Omega '\Subset I\times \mathbb {R}$ compactly containing $\textrm{sg}(u)\Delta \textrm{sg}(u+\psi )$ we have

$$\begin{aligned}  &   P_\varphi (\textrm{sg}(u),J\times \mathbb {R}) - P_\varphi (\textrm{sg}(u+\psi ),J\times \mathbb {R}) \\  &   \quad = P_\varphi (\textrm{sg}(u),\Omega ') - P_\varphi (\textrm{sg}(u+\psi ),\Omega ') \le \Lambda |\textrm{sg}(u)\Delta \textrm{sg}(u+\psi )|. \end{aligned}$$

By Lemma 2.4 and the equality

$$ \int _J |u-v|ds= |[\textrm{sg}(u)\Delta \textrm{sg}(v)]\cap [J\times \mathbb {R}]|, $$

the inequality above is equivalent to

$$ \mathscr {A}_\varphi (u,J) -\mathscr {A}_\varphi (u+\psi ,J) \le \Lambda \int _J|(u+\psi )-u|ds= \Lambda \int _J|\psi |ds, $$

and hence *u* is a Λ-local minimizer of $\mathscr {A}_\varphi$ in *I*.

Conversely, assume that φ is partially monotone and *u* is a Λ-local minimizer of $\mathscr {A}_\varphi$ in *I*. Let $F\in BV_{\textrm{loc}}(I\times \mathbb {R};\{0,1\})$ be such that $\textrm{sg}(u)\Delta F\Subset J\times (a,b)\Subset I\times \mathbb {R}$ for some $J\in \mathscr {O}_b(I)$ and $a,b\in \mathbb {R}.$ Let *v* be the function, whose subgraph is the vertical rearrangement of *F*,  i.e.,

$$ v(s) = a + \mathcal {H}^1(\{x_2\in (a,b):\,\, (s,x_2)\in F\}),\quad s\in I. $$

Notice that by the definition of the rearrangement, for a.e. s∈I, *v*(*s*) satisfies

$$\begin{aligned} |u(s)-v(s)| = \mathcal {H}^1((\textrm{sg}(u)\Delta F)\cap \{x_1=s\}) \end{aligned}$$

so that by the Fubini-Tonelli’s theorem,

2.3 $$\begin{aligned}  &   \int _{J'}|u-v|ds= \int _{J'} \mathcal {H}^1\big ( (\textrm{sg}(u)\Delta F)\cap \{x_1=s\}\big )ds\nonumber \\    &   = |(\textrm{sg}(u)\Delta F) \cap (J'\times \mathbb {R})|\quad \text {for any } J'\in \mathscr {O}_b(I). \end{aligned}$$

Repeating the same arguments of [4, Sect. 4] (see also [18]) we can show that $v\in BV_{\textrm{loc}}^1(I),$ $\mathrm {supp\,}(u-v)\Subset J,$

2.4 $$\begin{aligned} \mathcal {L}^1 (J') \le \int _{J'\times \mathbb {R}}|\left\langle D\chi _F,\, \textbf{e}_2\right\rangle |\quad \text {and}\quad \int _{J'}|Dv| \le \int _{J'\times \mathbb {R}}|\left\langle D\chi _F,\, \textbf{e}_1\right\rangle |\quad \text {for any } J'\in \mathscr {O}_b(I), \end{aligned}$$

where the Radon measures $-\left\langle D\chi _F,\, \textbf{e}_1\right\rangle$ and $-\left\langle D\chi _F,\, \textbf{e}_2\right\rangle$ are the horizontal and vertical components of $D\chi _F,$ which coincide with  and  respectively. Since $\varphi ^o$ is partially monotone, by (2.4)

2.5 $$\begin{aligned} \mathscr {A}_\varphi (v,J) =&\int _{J'} \varphi ^o(-Dv,1) = \int _{J'} \varphi ^o(|Dv|,\mathcal {L}^1) \le \int _{J'\times \mathbb {R}} \varphi ^o\big (|\left\langle D\chi _F,\, \textbf{e}_1\right\rangle |,|\left\langle D\chi _F,\, \textbf{e}_2\right\rangle |\big ) \nonumber \\ =&\int _{J'\times \mathbb {R}} \varphi ^o\big (\left\langle D\chi _F,\, \textbf{e}_1\right\rangle , \left\langle D\chi _F,\, \textbf{e}_2\right\rangle \big ) = \int _{J'\times \mathbb {R}} \varphi ^o(D\chi _F) = P_\varphi (F,J'\times \mathbb {R}) \end{aligned}$$

for all $$J'\in \mathscr {O}_b(I).$$

Now, by Lemma 2.4 and the Λ-local minimality of *u*,

2.6 $$\begin{aligned} P_\varphi (\textrm{sg}(u),J\times \mathbb {R}) - P_\varphi (\textrm{sg}(v),J\times \mathbb {R}) = \mathscr {A}_\varphi (u,J) - \mathscr {A}_\varphi (v,J) \le \Lambda \int _J |u-v|ds. \end{aligned}$$

Applying (2.3) and (2.5) with $J^{\prime }=J$ and recalling that $$\textrm{sg}(u)\Delta F \Subset J\times (a,b),$$ from (2.6) we conclude

$$\begin{aligned}&P_\varphi (\textrm{sg}(u),J\times (a,b)) - P_\varphi (F,J\times (a,b)) \\&\quad = P_\varphi (\textrm{sg}(u),J\times \mathbb {R}) - P_\varphi (F,J\times \mathbb {R}) \\&\quad \le P_\varphi (\textrm{sg}(u),J\times \mathbb {R}) - P_\varphi (\textrm{sg}(v),J\times \mathbb {R}) \le \Lambda |\textrm{sg}(u)\Delta F|. \end{aligned}$$

Thus, by definition, $\textrm{sg}(u)$ is a Λ-local minimizer of $P_\varphi$ in $I\times \mathbb {R}.$ □

Note that if φ is partially monotone, then $\mathscr {A}_\varphi (u,\cdot )=\mathscr {A}_\varphi (-u,\cdot ),$ and hence *u* is Λ-local minimizer if and only if so is -u. Thus, from Proposition 2.7 we get the following corollary.

#### Corollary 2.8

Let $u\in BV_{\textrm{loc}}(I).$ For any partially monotone anisotropy φ, the following assertions are equivalent:

- *u* is a Λ-local minimizer of $\mathscr {A}_\varphi$ in *I*; 
-u is a Λ-local minimizer of $\mathscr {A}_\varphi$ in *I*; 
- The subgraph $\textrm{sg}(u)$ of *u* is a Λ-local minimizer of $P_\varphi$ in $I\times \mathbb {R};$
- The (strict) epigraph $\textrm{epi}(u):=\{(s,t)\in I\times \mathbb {R}:\,\, u(s)<t\}$ is a Λ-local minimizer of $P_\varphi$ in $I\times \mathbb {R}.$

### Density estimates

The proof of the next lemma is well-known in the literature (see e.g. [8, 13]) and can be proved, for instance, using the filling-in or cutting-out with balls.

#### Lemma 2.9

Let be given an anisotropy φ on $\mathbb {R}^2$, $\Lambda \ge 0$ and an open set $\Omega \subseteq \mathbb {R}^2.$ Then for any $\Omega '\Subset \Omega$ there exist constants $r_0:=r_0(\varphi ,\Lambda ,\textrm{dist}(\partial \Omega ',\partial \Omega ))>0$ and $q_0:=q_0(\varphi ,\Lambda )\in (0,1/2)$ such that, for any Λ-local minimizer $E\in BV_{\textrm{loc}}(\Omega ;\{0,1\})$ of $P_\varphi$ in Ω, with $E=E^{(1)}$ (i.e., *E* coincides with its Lebesgue points),

$$\begin{aligned} P(E,B_r(x)) \le \frac{r}{q_0},\quad x\in \Omega ',\,\,\,r\in (0,r_0), \end{aligned}$$

$$\begin{aligned} q_0 \le \frac{|E\cap B_r(x)|}{|B_r(x)|} \le 1-q_0,\quad x\in \partial E,\,\,\, r\in (0,r_0), \end{aligned}$$

and

$$\begin{aligned} P(E,B_r(x)) \ge q_0 r,\quad x\in \partial E,\,\,\, r\in (0,r_0). \end{aligned}$$

From Lemma 2.9 and a covering argument we immediately deduce that every Λ-local minimizer $E=E^{(1)}$ in Ω satisfies

2.7 $$\begin{aligned}  &   \partial E=\overline{\partial ^*E},\quad \mathcal {H}^1\big (\Omega '\cap (\partial E\setminus \partial ^*E) \big ) = 0 \nonumber \\    &   \quad \text {and}\quad \mathcal {H}^1\big (\Omega '\cap (\overline{E}\setminus \mathring{E})\big ) <+\infty \quad \text {for any open } \Omega '\Subset \Omega . \end{aligned}$$

In particular, possibly changing a negligible set, *E* can be assumed open or closed.

#### Remark 2.10

Any Λ-local minimizer *E* of $P_\varphi$ in Ω satisfies

$$ P_\varphi (E,B_\rho (x)) \le P_\varphi (F,B_\rho (x)) + \Lambda \sqrt{\pi } \rho \sqrt{|E\Delta F|} $$

whenever $x\in \partial E,$ $B_\rho (x)\Subset \Omega$ and $E\Delta F\Subset B_\rho (x).$ Thus, *E* is ω-minimal in the sense of [20] with $\omega (\rho )=\Lambda \sqrt{\pi }\rho .$ In particular, by [20, Theorem 3.4], the set Σ of all points $x\in \Omega \cap \partial E$ around which $\Omega \cap \partial E$ is not a Lipschitz graph is discrete and is empty if $W^\varphi$ is not a quadrilateral^1^.

## Classification of local minimizers

In this section we classify the minimizers of $\mathscr {A}_\varphi ,$ i.e., study functions $u\in BV_{\textrm{loc}}(I)$ satisfying $\mathscr {A}_\varphi (u,J)\le \mathscr {A}_\varphi (v,J)$ for any open set J⋐I and $v\in BV_{\textrm{loc}}(I)$ with $\mathrm {supp\,}(u-v)\Subset J.$

### Theorem 3.1

(Characterization of local minimizers) Let φ be a partially monotone anisotropy, $I\subseteq \mathbb {R}$ be an interval and $u\in BV_{\textrm{loc}}(I).$ Let $\Gamma _u:=(I\times \mathbb {R})\cap \overline{\partial ^*\textrm{sg}(u)}$ be the generalized graph of *u* and $\nu _{\textrm{sg}(u)}:\Gamma _u\rightarrow \mathbb {S}^1$ be the unit normal field, outer to $(I\times \mathbb {R})\cap \textrm{sg}(u),$ defined $\mathcal {H}^1$-a.e. on $\Gamma _u.$ Then *u* is a local minimizer of $\mathscr {A}_\varphi$ in *I* if and only if there exists a vector $N\in \mathbb {R}^2$ such that

3.1 $$\begin{aligned} \varphi (N)=1\qquad \text {and}\qquad \left\langle N,\, \nu _{\textrm{sg}(u)}\right\rangle = \varphi ^o(\nu _{\textrm{sg}(u)})\quad \mathcal {H}^1 \text {-a.e. on } \Gamma _u. \end{aligned}$$

Moreover, *u* is monotone in *I*.

In the literature the vector *N* satisfying (3.1) is sometimes called a Cahn-Hoffman vector field associated to the rectifiable curve $\Gamma _u.$

### Proof

We expect this result to be well-known in the literature; for completeness we provide the proof.

⇐ We apply a calibration argument as in [4, Example 2.4]. Assume that there exists a vector *N* satisfying (3.1) and let $F\in BV_{\textrm{loc}}(I\times \mathbb {R};\{0,1\})$ be such that $F\Delta \textrm{sg}(u)\Subset J\times \mathbb {R}$ for some J⋐I. Then

$$\begin{aligned} P_\varphi (F,J\times \mathbb {R})&= \int _{(J\times \mathbb {R})\cap \partial ^*F} \varphi ^o(\nu _F)d\mathcal {H}^1 \ge \int _{(J\times \mathbb {R})\cap \partial ^*F} \left\langle \nu _F,\, N\right\rangle \,d\mathcal {H}^1. \end{aligned}$$

On the other hand, by the divergence theorem

$$\begin{aligned} 0= &   \int _{F\setminus \textrm{sg}(u)} \textrm{div}N\,dx- \int _{\textrm{sg}(u)\setminus F} \textrm{div}N\,dx\\= &   \int _{(J\times \mathbb {R})\cap \partial ^*F} \left\langle \nu _F,\, N\right\rangle \,d\mathcal {H}^1 - \int _{(J\times \mathbb {R})\cap \partial ^*\textrm{sg}(u)} \left\langle \nu _{\textrm{sg}(u)},\, N\right\rangle \,d\mathcal {H}^1, \end{aligned}$$

and thus

$$\begin{aligned}&\int _{(J\times \mathbb {R})\cap \partial ^*F} \left\langle \nu _F,\, N\right\rangle \,d\mathcal {H}^1 =\int _{(J\times \mathbb {R})\cap \partial ^*\textrm{sg}(u)} \left\langle \nu _{\textrm{sg}(u)},\, N\right\rangle \,d\mathcal {H}^1 \\&= \int _{(J\times \mathbb {R})\cap \partial ^*\textrm{sg}(u)} \varphi ^o(\nu _{\textrm{sg}(u)})\,d\mathcal {H}^1 =P_\varphi (\textrm{sg}(u),J\times \mathbb {R}). \end{aligned}$$

Hence $\textrm{sg}(u)$ is a local minimizer of $P_\varphi$ in $I\times \mathbb {R}.$ Then Corollary 2.8 implies that *u* is a local minimizer of $\mathscr {A}_\varphi$ in *I*.

⇒ Assume that *u* is a local minimizer of $\mathscr {A}_\varphi$ in *I*. Since |Du|(J)<+∞ for any open interval J⋐I, we have $u\in L^\infty (J).$ In particular, $u\in BV_{\textrm{loc}}(I).$

Let J⋐I be any interval, whose boundary points are not on the jump set of *u*;  such an interval exists because *u* has at most countably many jumps. Let *v* be the function such that v=u in I\J and linear in *J* such that the traces of *u* and *v* on ∂J coincide.

Let *p* and *q* be the endpoints of the graph of *v* corresponding to the endpoints of *J*, so that the graph is the line segment [*p*, *q*].

By the local boundedness of *u*,  $\textrm{sg}(u)\Delta \textrm{sg}(v)\Subset I\times \mathbb {R}.$ Then by the local minimality of *u* and the anisotropic minimality of segments [10],

$$ P_\varphi (\textrm{sg}(u),J\times \mathbb {R}) \ge P_\varphi (\textrm{sg}(v),J\times \mathbb {R}) \ge P_\varphi (\textrm{sg}(u),J\times \mathbb {R}), $$

and hence $P_\varphi (\textrm{sg}(u),J\times \mathbb {R}) = P_\varphi (\textrm{sg}(v),J\times \mathbb {R}).$ Choose a vector $N\in \mathbb {R}^2$ satisfying φ(N)=1 and $\left\langle \nu _{[p,q]},\, N\right\rangle =\varphi ^o(\nu _{[p,q]})$ on [*p*, *q*]. As above, by the divergence formula

$$\begin{aligned} 0= &   \int _{\textrm{sg}(v)\setminus \textrm{sg}(u)} \textrm{div}N\,dx- \int _{\textrm{sg}(u)\setminus \textrm{sg}(v)} \textrm{div}N\,dx\\ \\  = &   \int _{(J\times \mathbb {R})\cap \partial ^*\textrm{sg}(v)} \left\langle \nu _F,\, N\right\rangle \,d\mathcal {H}^1 - \int _{(J\times \mathbb {R})\cap \partial ^*\textrm{sg}(u)} \left\langle \nu _{\textrm{sg}(u)},\, N\right\rangle \,d\mathcal {H}^1. \end{aligned}$$

Thus,

3.2 $$\begin{aligned}  &   P_\varphi (\textrm{sg}(v),J\times \mathbb {R}) \nonumber \\  &   \quad = P_\varphi (\textrm{sg}(u),J\times \mathbb {R}) = \int _{(J\times \mathbb {R})\cap \partial ^*\textrm{sg}(u)} \varphi ^o(\nu _{\textrm{sg}(u)})d\mathcal {H}^1 \ge \int _{(J\times \mathbb {R})\cap \partial ^*\textrm{sg}(u)} \left\langle \nu _F,\, N\right\rangle \,d\mathcal {H}^1 \nonumber \\  &   \quad =\int _{(J\times \mathbb {R})\cap \partial ^*\textrm{sg}(v)} \left\langle \nu _{\textrm{sg}(v)},\, N\right\rangle \,d\mathcal {H}^1 = \int _{(J\times \mathbb {R})\cap \partial ^*\textrm{sg}(v)} \varphi ^o(\nu _{\textrm{sg}(v)})\,d\mathcal {H}^1 =P_\varphi (\textrm{sg}(v),J\times \mathbb {R}), \end{aligned}$$

where in the fourth equality we used that *v* is linear in *J*. Thus, all inequalities in (3.2) are in fact equalities. Since $\varphi ^o(\nu _{\textrm{sg}(u)}) \ge \left\langle \nu _{\textrm{sg}(u)},\, N\right\rangle$ $\mathcal {H}^1$-a.e. on $(J\times \mathbb {R})\cap \partial ^*\textrm{sg}(u)$ and

$$ \int _{(J\times \mathbb {R})\cap \partial ^*\textrm{sg}(u)} \varphi ^o(\nu _{\textrm{sg}(u)})d\mathcal {H}^1 = \int _{(J\times \mathbb {R})\cap \partial ^*\textrm{sg}(u)} \left\langle \nu _{\textrm{sg}(u)},\, N\right\rangle \,d\mathcal {H}^1, $$

from the Chebyshev inequality it follows that $\varphi ^o(\nu _{\textrm{sg}(u)}) = \left\langle \nu _{\textrm{sg}(u)},\, N\right\rangle$ $\mathcal {H}^1$-a.e. on $(J\times \mathbb {R})\cap \partial ^*\textrm{sg}(u).$ Now, consider a sequence $J_k\nearrow I$ of open relatively compact intervals with $J_k\cap J_u = \emptyset$ and the associated constant vectors $N_k\in \partial W^\varphi .$ Notice that each $N_k$ satisfies

3.3 $$\begin{aligned} \varphi (N_k)=1\qquad \text {and}\qquad \varphi ^o(\nu _{\textrm{sg}(u)}) = \left\langle \nu _{\textrm{sg}(u)},\, N_k\right\rangle \quad \mathcal {H}^1 \text {-a.e. on } (J_k\times \mathbb {R})\cap \partial ^*\textrm{sg}(u). \end{aligned}$$

Since $\partial W^\varphi$ is compact, there is no loss of generality in assuming $N_k\rightarrow N$ for some $N\in \partial W^\varphi .$ Notice that, given $\bar{k} \in \mathbb {N}$, all $N_k$ with $k\ge \bar{k}$ satisfy (3.3) in $J_{\bar{k}}.$ Since $N_k$ appear linearly in the second relation of (3.3), it follows that any vector in the closed convex hull $K_{\bar{k}}$ of $\cup _{k\ge \bar{k}}N_k$ also satisfies (3.3). Clearly, *N* belongs to $K_{\bar{k}}$ for all $\bar{k}.$ As $J_k\nearrow I,$ it follows that *N* satisfies (3.1).

Finally, let us show that *u* is monotone, i.e., it admits a monotone representative. Indeed, suppose that there exist (a,b)⋐I and $t\in \mathbb {R}$ such that $(a,t),(b,t)\in \Gamma _u.$ Let us define the competitor $v = u\chi _{I\setminus (a,b)} + t\chi _{(a,b)}.$ By the local minimality of *u*,  for any open interval *J* with $(a,b)\Subset J\Subset I$ we have

3.4 $$\begin{aligned}  &   0\le \mathscr {A}_\varphi (v,J) - \mathscr {A}_\varphi (u,J) = \int _{(a,b)} \Big (\varphi ^o(0,1)d\mathcal {L}^1 - \varphi ^o(-Du,1)\Big ) \nonumber \\  &   \quad \quad \quad + \varphi ^o(\textbf{e}_1)\Big ( |v^+(a)-v^-(a)| - |u^+(a)-u^-(a)| + |v^+(b)-v^-(b)| - |u^+(b)-u^-(b)|\Big ), \end{aligned}$$

where in the equality we used (2.2). By the definition of *v* and the choice of *t*,  $u^-(a)=v^-(a),$ $u^+(b)=v^+(b),$ $v^+(a)=v^-(b)=t$ and

$$ |v^+(a)-v^-(a)| \le |u^+(a)-u^-(a)|,\qquad |v^+(b)-v^-(b)| \le |u^+(b)-u^-(b)|. $$

Moreover, by the partial monotonicity of $\varphi ^o$ we have

$$ \int _a^b \varphi ^o(-u',1)ds\ge \int _a^b \varphi ^o(0,1)ds, $$

and hence, by (3.4) and (2.2) we have

$$\begin{aligned}  &   0\le \varphi ^o(\textbf{e}_1)\Big (|u^+(a)-u^-(a)| - |v^+(a)-v^-(a)| + |u^+(b)-u^-(b)| -|v^+(b)-v^-(b)|\Big ) \\  &   \quad \le \int _a^b \varphi ^o(0,1)ds- \int _a^b \varphi ^o(-u',1)ds- \varphi ^o(D^su,0)(a,b) \le 0. \end{aligned}$$

Thus, all inequalities are in fact equalities, $$u^+(a)=u^-(b)=t,$$ $u^{\prime }=0$ a.e. in (*a*, *b*) and $$D^su=0.$$ This implies u=v in (*a*, *b*). This observation shows that for any $$\lambda \in \mathbb {R}$$ the set $$\{u=\lambda \}$$ is either empty, or one point or an interval. Therefore, *u* is monotone. □

### Example 3.2

(Strictly convex anisotropies) Assume that $\varphi ^o$ is strictly convex, i.e.,

$$ \varphi ^o(x+y)<\varphi ^o(x)+\varphi ^o(y)\quad \text {whenever } |x|=|y| \text { with } x\ne \pm y. $$

Then for any interval $I\subseteq \mathbb {R},$ the function $u\in BV_{\textrm{loc}}(I)$ is a local minimizer of $\mathscr {A}_\varphi$ if and only if *u* is linear. Indeed, by the strict convexity of φ, for any $N\in \partial W^\varphi$ there exists a unique $\nu \in \mathbb {S}^1$ such that $\left\langle N,\, \nu \right\rangle = \varphi ^o(\nu ).$ Thus, by Theorem 3.1*u* is a local minimizer of $\mathscr {A}_\varphi$ in *I* if and only if $\Gamma _u$ admits a constant unit normal $\mathcal {H}^1$-a.e., which is equivalent to say that *u* is linear.

### Example 3.3

(Square anisotropy) Let $W^\varphi =[-1,1]^2$ and $I\subseteq \mathbb {R}$ be an interval, Then *u* is a local minimizer of $\mathscr {A}_\varphi$ if and only if *u* is monotone. Indeed, by Theorem 3.1 every local minimizer is monotone. Conversely, consider any nondecreasing function $u:I\rightarrow \mathbb {R}.$ By monotonicity, the unit normals $\nu _u$ to $\Gamma _u$ lie in the smaller closed arc of $\mathbb {S}^1$ between $-\textbf{e}_1$ (jump part) and $\textbf{e}_2$ (constant part). Thus, any constant vector $N=(-1,1)\in \partial W^\varphi$ satisfies

$$ \left\langle N,\, \nu _u\right\rangle = |\left\langle \nu _u,\, \textbf{e}_1\right\rangle |+ |\left\langle \nu _u,\, \textbf{e}_2\right\rangle | = \varphi ^o(\nu _u)\quad \mathcal {H}^1 \text {-a.e. on } \Gamma _u. $$

Hence, by Theorem 3.1, *u* is a local minimizer.

### Example 3.4

(Lens-shaped anisotropies) Given a>0, let $$\gamma \in C^1([-a,0])$$ be a strictly increasing concave function with γ(-a)=0 and $\gamma^{\prime }\left(0\right)=0.$ Let $$W^\varphi $$ be the convex set symmetric with respect to the coordinate axes such that $$((-\infty ,0)\times (0,+\infty ))\cap \partial W^\varphi $$ is the graph of γ. Let $$I\subseteq \mathbb {R}$$ be an interval. Then $$u\in BV_{\textrm{loc}}(I)$$ is a local minimizer of $$\mathscr {A}_\varphi $$ in *I* if and only if either *u* is linear, or *u* is monotone and piecewise linear, and all segments/half-lines of its graph are tangent^2^ to $$W^\varphi $$ at exactly one of the two points $$\pm \frac{\textbf{e}_1}{\varphi (\textbf{e}_1)}.$$

## Regularity of Λ-minimizers

Now consider the case Λ>0. In this case a general characterization of Λ-local minimizers as in Theorem 3.1 seems not available. In this section, under some assumptions of φ, we show that if the $L^\infty$-norm of a Λ-minimizer of $\mathscr {A}_\varphi$ in *I* is sufficiently small, then *u* is Lipschitz continuous in *I*.

### Theorem 4.1

(Regularity of Λ-minimizers) Let φ be a partially monotone anisotropy such that $W^\varphi$ does not have vertical facets (so that $\pm \textbf{e}_1$ is an “outer normal” to $W^\varphi$ only at $\frac{\pm \textbf{e}_1}{\varphi (\textbf{e}_1)}$). Given a bounded open interval $I\subset \mathbb {R}$ and Λ>0, let $u\in BV_{\textrm{loc}}(I)$ be a Λ-local minimizer of $\mathscr {A}_\varphi$ in *I* satisfying

4.1 $$\begin{aligned} \Vert u\Vert _\infty < \min \Big \{\tfrac{\alpha _0\varphi (\textbf{e}_1)}{4\Lambda }, \tfrac{\alpha _0}{2\Lambda \varphi (\textbf{e}_2)}, \, \tfrac{|I|\varphi (\textbf{e}_1)}{4\Lambda \varphi (\textbf{e}_2)}\Big \}, \end{aligned}$$

with

4.2 $$\begin{aligned} \alpha _0=\alpha _0(\varphi ):=\tfrac{P_\varphi (W^\varphi )}{(2P_\varphi (W^\varphi )+ 1) \,\sqrt{|W^\varphi |} }>0. \end{aligned}$$

Then *u* is Lipschitz continuous in *I*. Moreover, if φ is $$C^2$$ and elliptic, then $$u\in C^{1,1}(I),$$ that is, *u* is continuously differentiable and its derivative $u^{\prime }$ is Lipschitz continuous in *I*.

Note that every partially monotone elliptic anisotropy satisfies the assumption of the theorem. Moreover, by Remark 2.6, u∈BV(I). Furthermore, by the partial monotonicity of φ and Corollary 2.8, the subgraph and epigraph of *u* are Λ-local minimizers of $P_\varphi$ in $I\times \mathbb {R}.$ Notice also that when $W^\varphi$ is not a quadrilateral, Remark 2.10 ensures that the boundaries of these sets in $I\times \mathbb {R}$ are locally given by a Lipschitz graph. However, this graphicality property of $\textrm{sg}(u)$ and $\textrm{epi}(u)$ does not yield that *u* itself is Lipschitz; for instance, *u* may have jump discontinuities (see the function $u_{a,b}$ in (1.4)).

The proof of Theorem 4.1 is postponed to the end of the section after some ancillary results. We start with the following property of Wulff shapes.

### Lemma 4.2

Let a bounded set $D\in BV(\mathbb {R}^2;\{0,1\})$ and a Wulff shape $W_r^\varphi$ with r>0 be such that $\mathring{W}_r^\varphi \cap D=\emptyset$ and $\mathcal {H}^1((\partial ^* D)\cap \partial W_r^\varphi )>0.$ Then

4.3 $$\begin{aligned} \int _{(\partial ^*D)\setminus \partial ^* W_r^\varphi }\varphi ^o(\nu _D)\,d\mathcal {H}^1 - \int _{(\partial ^*D)\cap \partial ^* W_r^\varphi } \varphi ^o(\nu _D)\,d\mathcal {H}^1 \ge \alpha _0 \, \min \Big \{\sqrt{|D|},\frac{|D|}{r}\Big \}, \end{aligned}$$

where $\alpha _0>0$ is given in (4.2).

### Proof

The proof is similar to [10, Proposition 6.3]. Recall that the isoperimetric inequality (see e.g. [9]) says

4.4 $$\begin{aligned} P_\varphi (E) \ge c_\varphi \,\sqrt{|E|},\quad E\in BV(\mathbb {R}^2;\{0,1\}), \end{aligned}$$

where $c_\varphi :=\frac{P_\varphi (W^\varphi )}{\sqrt{|W^\varphi |}},$ and the equality in (4.4) holds if and only if $E = x+ rW^\varphi =W_r^\varphi (x)$ for some $x\in \mathbb {R}^2$ and r≥0. When φ is Euclidean, $c_\varphi =\sqrt{4\pi }.$

Fix any α>1. First consider the case

4.5 $$\begin{aligned} \int _{\partial ^*D\setminus \partial ^* W_r^\varphi }\varphi ^o(\nu _D)\,d\mathcal {H}^1 \le \alpha \int _{\partial ^*D\cap \partial ^* W_r^\varphi } \varphi ^o(\nu _D)\,d\mathcal {H}^1 \end{aligned}$$

Since $\mathring{W}_r^\varphi \cap D=\emptyset ,$ by [15, Theorem 16.3] one has $\partial ^*(D\cup W_r^\varphi ) \approx _{\mathcal {H}^1} [(\partial ^*D){\setminus } W_r^\varphi ] \cup [(\partial ^*W_r^\varphi ){\setminus } \partial ^*D],$ where $A\approx _\mu B$ stands for $\mu (A\Delta B)=0.$ Therefore,

4.6 $$\begin{aligned}&\int _{(\partial ^*D) {\setminus } \partial ^* W_r^\varphi }\varphi ^o(\nu _D)\,d\mathcal {H}^1 - \int _{(\partial ^*D)\cap \partial ^* W_r^\varphi } \varphi ^o(\nu _D)\,d\mathcal {H}^1 \nonumber \\&\quad = \int _{\partial ^*(D\cup W_r^\varphi )}\varphi ^o(\nu _{D\cup W_r^\varphi })\,d\mathcal {H}^1 - \int _{\partial ^* W_r^\varphi } \varphi ^o(\nu _{W_r^\varphi })\,d\mathcal {H}^1 \nonumber \\&\quad = P_\varphi (D\cup W_r^\varphi ) - P_\varphi (W_r^\varphi ) \ge c_\varphi \Big (\sqrt{|D\cup W_r^\varphi |} - \sqrt{|W_r^\varphi |}\Big ) = \tfrac{c_\varphi |D|}{\sqrt{|D\cup W_r^\varphi |} + \sqrt{|W_r^\varphi |}}, \end{aligned}$$

where in the first inequality we used the isoperimetric inequality (4.4). Moreover, by (4.5) and again by the isoperimetric inequality and the assumption $\mathring{W}_r^\varphi \cap D=\emptyset ,$

$$\begin{aligned} c_\varphi \sqrt{|D\cup W_r^\varphi |}&\le P_\varphi (D\cup W_r^\varphi ) = \int _{(\partial ^*D){\setminus } \partial W_r^\varphi }\varphi ^o(\nu _D)\,d\mathcal {H}^1 + \int _{(\partial ^*W_r^\varphi ){\setminus } \partial ^* D} \varphi ^o(\nu _{W_r^\varphi })\,d\mathcal {H}^1 \\&\le \alpha \int _{(\partial ^*D)\cap \partial ^* W_r^\varphi } \varphi ^o(\nu _D)\,d\mathcal {H}^1 + \int _{(\partial ^*W_r^\varphi ){\setminus } \partial D} \varphi ^o(\nu _{W_r^\varphi })\,d\mathcal {H}^1 \\&\le \alpha \int _{\partial ^*W_r^\varphi } \varphi ^o(\nu _{W_r^\varphi })d\mathcal {H}^1 = \alpha P_\varphi (W_r^\varphi ). \end{aligned}$$

Thus, recalling $c_\varphi \sqrt{|W_r^\varphi |} = P_\varphi (W_r^\varphi ),$ from (4.6) we get

4.7 $$\begin{aligned} \int _{(\partial ^*D)\setminus \partial ^* W_r^\varphi }\varphi ^o(\nu _D)\,d\mathcal {H}^1 - \int _{(\partial ^*D)\cap \partial ^* W_r^\varphi } \varphi ^o(\nu _D)\,d\mathcal {H}^1 \ge \tfrac{c_\varphi |D|}{(\alpha +1)P_\varphi (W_r^\varphi )} = \tfrac{|D|}{(\alpha +1)r\sqrt{|W^\varphi |} } . \end{aligned}$$

Now consider the case

4.8 $$\begin{aligned} \int _{(\partial ^*D)\setminus \partial ^* W_r^\varphi }\varphi ^o(\nu _D)\,d\mathcal {H}^1 > \alpha \int _{(\partial ^*D)\cap \partial ^* W_r^\varphi } \varphi ^o(\nu _D)\,d\mathcal {H}^1 \end{aligned}$$

so that

4.9 $$\begin{aligned} \int _{(\partial ^*D)\setminus \partial ^* W_r^\varphi }\varphi ^o(\nu _D)\,d\mathcal {H}^1 - \int _{(\partial ^*D)\cap \partial ^* W_r^\varphi } \varphi ^o(\nu _D)\,d\mathcal {H}^1 > \Big (1-\frac{1}{\alpha }\Big ) \int _{(\partial ^*D)\setminus \partial ^* W_r^\varphi }\varphi ^o(\nu _D)\,d\mathcal {H}^1. \end{aligned}$$

Then by the isoperimetric inequality, (4.9) and (4.8) we get

$$\begin{aligned} c_\varphi \,|D|^{1/2} \le P_\varphi (D)&= \int _{(\partial ^* D)\setminus \partial ^* W_r^\varphi }\varphi ^o(\nu _D)\,d\mathcal {H}^1 + \int _{(\partial ^*D)\cap \partial ^* W_r^\varphi }\varphi ^o(\nu _D)\,d\mathcal {H}^1 \\&\le \Big (1 + \frac{1}{\alpha }\Big )\,\int _{(\partial ^* D)\setminus \partial ^* W_r^\varphi }\varphi ^o(\nu _D)\,d\mathcal {H}^1 \le \frac{\alpha +1}{\alpha -1}\,\Big ( \int _{(\partial ^*D) \setminus \partial ^* W_r^\varphi }\varphi ^o(\nu _D)\,d\mathcal {H}^1 \\&\quad - \int _{(\partial ^*D)\cap \partial ^* W_r^\varphi } \varphi ^o(\nu _D)\,d\mathcal {H}^1 \Big ). \end{aligned}$$

Combining this inequality with (4.7) we deduce

$$ \int _{(\partial ^*D){\setminus } \partial ^* W_r^\varphi }\varphi ^o(\nu _D)\,d\mathcal {H}^1 - \int _{(\partial ^*D)\cap \partial ^* W_r^\varphi } \varphi ^o(\nu _D)\,d\mathcal {H}^1 \ge \min \Big \{ \tfrac{(\alpha -1)c_\varphi \sqrt{|D|}}{\alpha +1}, \tfrac{|D|}{(\alpha +1)r\sqrt{|W^\varphi |}}\Big \}. $$

Now, choosing $\alpha :=1+\frac{1}{P_\varphi (W^\varphi )}$ we get (4.3). □

Next we “improve” the regularity of the subgraph and the epigraph of Λ-minimizers *u*. For simplicity, let $E_u$ and $F_u$ be the open representatives of $\textrm{sg}(u)$ and $\textrm{epi}(u),$ respectively (see Lemma 2.9), and let

$$ \Gamma _u:=(I\times \mathbb {R})\cap \partial E_u=(I\times \mathbb {R})\cap \partial F_u $$

be the (generalized) graph of *u* in *I*. By Remark 2.10$\Gamma _u$ is a locally Lipschitz curve^3^ (thus an arcwise connected set) and, as the traces of *u* on ∂I are well-defined (see Remark 2.6), its topological closure $\overline{\Gamma }_u$ consists of the union of $\Gamma _u$ and two points on ∂I, whose vertical coordinates correspond to the traces of *u*.

### Proposition 4.3

(Contact φ-ball condition) Let φ be a partially monotone anisotropy on $\mathbb {R}^2$, $I\subset \mathbb {R}$ be a bounded open interval, Λ>0 and $u\in BV_{\textrm{loc}}(I)$ be a Λ-local minimizer of $\mathscr {A}_\varphi$ in *I* with

4.10 $$\begin{aligned} \Vert u\Vert _\infty \le \frac{\alpha _0\varphi (\textbf{e}_1)}{4\Lambda }, \end{aligned}$$

where $\alpha _0$ is given by (4.2). Then for any $r\in (0,\frac{\alpha _0}{\Lambda }):$

- if $\mathring{W}_r^\varphi (y)\cap F_u=\emptyset$ with $\Gamma _u\cap \partial W_r^\varphi (y)\ne \emptyset ,$ then $\overline{\Gamma }_u \cap \partial W_r^\varphi (y)$ is connected (possibly a singleton);
- if $\mathring{W}_r^\varphi (z)\cap E_u=\emptyset$ with $\Gamma _u\cap \partial W_r^\varphi (z)\ne \emptyset ,$ then $\overline{\Gamma }_u \cap \partial W_r^\varphi (z)$ is connected (possibly a singleton);
- for any $x\in \Gamma _u$ there exist φ-balls $W_r^\varphi (y)$ and $W_r^\varphi (z)$ such that $\mathring{W}_r^\varphi (y)\cap E_u=\emptyset ,$ $\mathring{W}_r^\varphi (z)\cap F_u=\emptyset ,$ and $\Gamma _u\cap \partial W_r^\varphi (y)$ and $\Gamma _u \cap \partial W_r^\varphi (z)$ are connected sets (possibly a singleton) containing *x*.

**Fig. 1:**
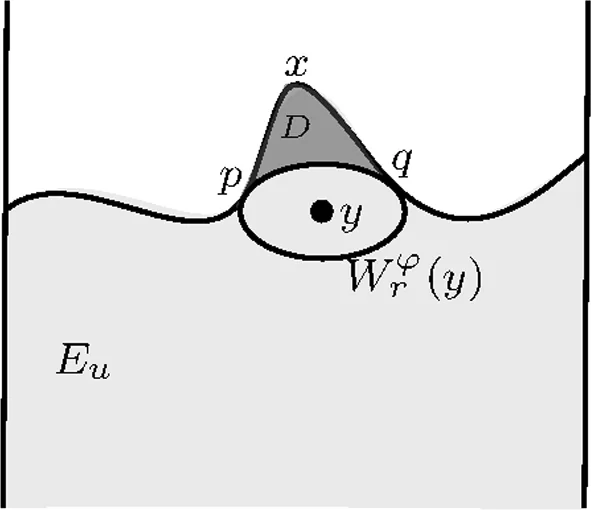
A contact Wulff shape touching at two points

Notice that the φ-balls $\mathring{W}_r^\varphi (y)$ and $\mathring{W}_r^\varphi (z)$ need not to be contained in $I\times \mathbb {R}.$ The assertion (c) is related to the $rW^\varphi$-condition in the literature, see e.g. [3, Definition 5].

### Proof

(a) Assume by contradiction that there exist $r\in (0,\frac{\alpha _0}{\Lambda })$ and a φ-ball $W_r^\varphi (y)$ such that $\mathring{W}_r^\varphi (y)\cap F_u=\emptyset$ and the intersection $\overline{\Gamma }_u \cap \partial \mathring{W}_r^\varphi (y)$ is not connected. Let us denote by p≠q the leftmost/rightmost points of this intersection; in case there are several upmost left and/or upmost right points we select those with the smallest vertical coordinate. Note that $p,q\in \overline{I}\times \mathbb {R}.$

Let *D* be the nonempty open set enclosed by the subcurves of $\overline{\Gamma }_u$ and $\partial \mathring{W}_r^\varphi (y)$ between *p* and *q*,  not intersecting $\mathring{W}_r^\varphi (y),$ see Fig. 1. Since $W^\varphi$ is symmetric with respect to the coordinate axes, $y\pm r\frac{\textbf{e}_1}{\varphi (\textbf{e}_1)}$ are the upmost left and upmost right points of $W_r^\varphi (y)$ and hence,

$$ \left\langle y-r\tfrac{\textbf{e}_1}{\varphi (\textbf{e}_1)},\, \textbf{e}_1\right\rangle \le \min \{\left\langle p,\, \textbf{e}_1\right\rangle ,\left\langle q,\, \textbf{e}_1\right\rangle \} \le \max \{\left\langle p,\, \textbf{e}_1\right\rangle ,\left\langle q,\, \textbf{e}_1\right\rangle \} \le \left\langle y + r\tfrac{\textbf{e}_1}{\varphi (\textbf{e}_1)},\, \textbf{e}_1\right\rangle . $$

Thus, recalling that *p* and *q* lie on the graph of *u*, 

$$ D\subset \Big [\left\langle y,\, \textbf{e}_1\right\rangle -\tfrac{r}{\varphi (\textbf{e}_1)},\left\langle y,\, \textbf{e}_1\right\rangle + \tfrac{r}{\varphi (\textbf{e}_1)}\Big ]\times [-\Vert u\Vert _\infty ,\Vert u\Vert _\infty ] $$

and therefore

4.11 $$\begin{aligned} 0<|D| \le \frac{4\Vert u\Vert _\infty r}{\varphi (\textbf{e}_1)}. \end{aligned}$$

First assume that

$$ D\Subset I\times \mathbb {R}. $$

Then by Λ-minimality (Corollary 2.8), for any open set $\Omega '\Subset I\times \mathbb {R},$ compactly containing $D\subset E_u,$ we have

4.12 $$\begin{aligned} P_\varphi (E_u,\Omega ') \le P_\varphi (E_u\setminus D,\Omega ') + \Lambda |D|. \end{aligned}$$

Since $\mathring{W}_r^\varphi (y) \cap D =\emptyset$ and $\mathcal {H}^1((\partial ^*D)\cap \partial W_r^\varphi (y) )>0$ (because $W_r^\varphi (y)$ touches $\partial E_u$ at two different points), we can apply Lemma 4.2 to get

$$\begin{aligned}  &   P_\varphi (E_u,\Omega ') -P_\varphi (E_u\setminus D,\Omega ') \\  = &   \int _{(\partial ^*D)\setminus \partial ^* W_r^\varphi (y)}\varphi ^o(\nu _D)\,d\mathcal {H}^1 - \int _{(\partial ^*D)\cap \partial ^* W_r^\varphi (y)} \varphi ^o(\nu _D)\,d\mathcal {H}^1 \ge \alpha _0 \, \min \Big \{\sqrt{|D|},\frac{|D|}{r}\Big \}, \end{aligned}$$

and thus

4.13 $$\begin{aligned} \alpha _0 \, \min \Big \{\sqrt{|D|},\frac{|D|}{r}\Big \} \le \Lambda |D|. \end{aligned}$$

Now, if $\sqrt{|D|}\le \frac{|D|}{r},$ then by (4.13), (4.11) and the assumption $r<\frac{\alpha _0}{\Lambda }$ we have

$$ \alpha _0 \le \Lambda \sqrt{|D|} < \Lambda \sqrt{ \tfrac{4\Vert u\Vert _\infty \alpha _0 }{\Lambda \varphi (\textbf{e}_1)} }\qquad \text {so that}\qquad \Vert u\Vert _\infty > \tfrac{\alpha _0\varphi (\textbf{e}_1)}{4\Lambda }, $$

which contradicts (4.10). On the other hand, if $\frac{|D|}{r}<\sqrt{|D|},$ then again by (4.13) and the choice of *r*, 

$$ \frac{\alpha _0}{\Lambda } \le r<\frac{\alpha _0}{\Lambda }, $$

a contradiction.

In case

$$ D\cap ((\partial I)\times \mathbb {R}) \ne \emptyset , $$

we fix ϵ>0 and choose an interval J⋐I with $|I\setminus J|<\epsilon$ and replace *D* with $D_\epsilon :=D\cap ( J\times \mathbb {R}).$ Notice that for small ϵ, $D_\epsilon$ is non-empty and satisfies $\mathring{W}_r^\varphi (y) \cap D_\epsilon =\emptyset$ and $\mathcal {H}^1((\partial ^*D_\epsilon ) \cap \partial W_r^\varphi (y))>0.$ Thus, we can use $E_u{\setminus } D_\epsilon$ as a competitor in (4.12) to get (4.13) with $D_\epsilon$ in place of *D*. Now letting $\epsilon \rightarrow 0^+$ we conclude (4.13) and the remaining part of the contradictory argument runs as above.

(b) is proven as (a).

(c) Fix any $x\in \Gamma _u,$ $r\in (0,\frac{\alpha _0}{\Lambda })$ and consider the set

$$ S:=\{y\in \mathbb {R}^2:\,\,\textrm{dist}_\varphi (y,F_u)=\textrm{dist}_\varphi (y,\Gamma _u)=r\}. $$

Note that S contains two little arcs outside the strip $I\times \mathbb {R},$ and hence, tangent balls may have centers outside it. Observe also that for any y∈S the φ-ball $\mathring{W}_r^\varphi (y)$ is “tangent” to $\overline{\Gamma }_u$ at some point and does not intersect $F_u.$ In view of (a) and (b), it suffices to show that there exists $\bar{y}\in S$ such that $x\in \partial W_r^\varphi (\bar{y}).$ Indeed, otherwise, as $\Gamma _u$ is a generalized graph (an arcwise connected set), we could find y∈S such that $\Gamma _u \cap \partial W_r^\varphi (y)$ contains two distinct points *p* and *q*,  and *x* lies in the relative interior of the subcurve of $\Gamma _u$ with endpoints *p* and *q*,  but does not belong to $\partial W_r^\varphi (y).$ However, by (a), the set $\overline{\Gamma }_u \cap \partial W_r^\varphi (y)$ is connected, and hence $x\in \partial W_r^\varphi (y),$ a contradiction.

For a similar reason, for any $x\in \Gamma _u$ and $r\in (0,\frac{\alpha _0}{\Lambda })$ there exists $W_r^\varphi (y)$ such that $\mathring{W}_r^\varphi (y)\cap E_u=\emptyset$ and $x\in \Gamma _u \cap \partial W_r^\varphi (y).$ □

One corollary of Proposition 4.3 is the following Lipschitz regularity of *u*.

### Corollary 4.4

Let φ be a partially monotone anisotropy such that $W^\varphi$ does not have vertical facets and, given a bounded open interval $I\subset \mathbb {R}$ and Λ>0, let $u\in BV_{\textrm{loc}}(I)$ be a Λ-local minimizer of $\mathscr {A}_\varphi$ in *I* satisfying (4.1). Then *u* is Lipschitz continuous in *I*.

### Proof

For simplicity, suppose I=(-a,a) for some a>0. We claim that there exists λ>0 such that

4.14 $$\begin{aligned} \left\langle \nu _{E_u} ,\, \textbf{e}_2\right\rangle \ge \lambda \quad \mathcal {H}^1 \text {-a.e. on } (I\times \mathbb {R})\cap \partial ^* E_u, \end{aligned}$$

where $\nu _{E_u}$ is the generalized outer unit normal to $E_u.$ Indeed, by contradiction, assume that there exists a sequence $(x_k)\subset (I\times \mathbb {R})\cap \partial ^* E_u$ such that $\left\langle \nu _{E_u}(x_k),\, \textbf{e}_2\right\rangle \rightarrow 0.$ Possibly passing to a not relabelled subsequence, replacing *u* with -u and changing the orientation of *I* (i.e., using the mirror symmetry with respect to the vertical axis) if necessary, we may assume $(x_k)\subset (-a,0]\times \mathbb {R}$ and $\nu _{E_u}(x_k)\cdot \textbf{e}_1\rightarrow -1$ as $k\rightarrow +\infty .$

**Fig. 2:**
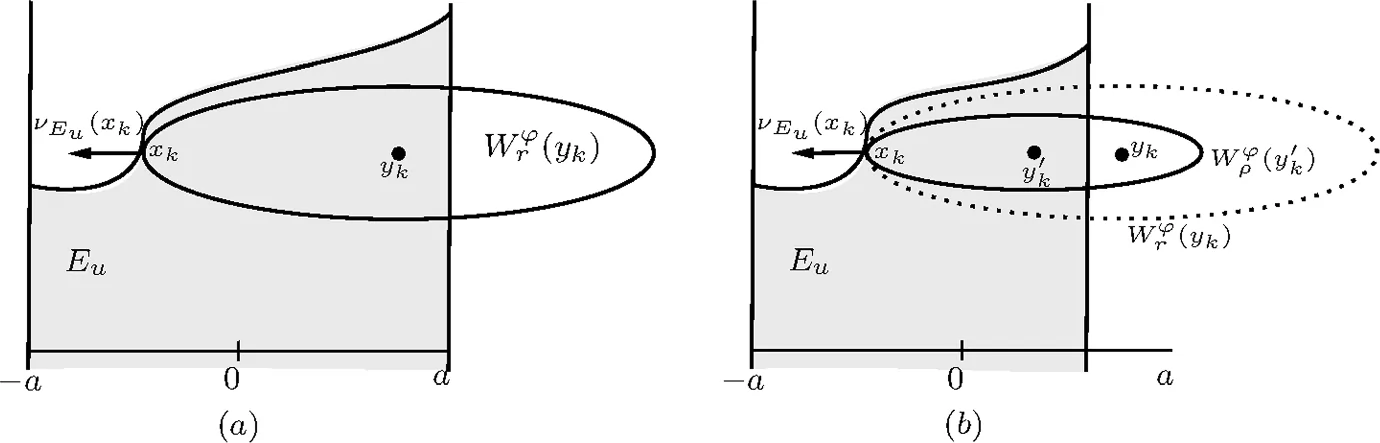
A contact Wulff shapes at the vertical part of the graph of u

Given $\epsilon \in (0,1),$ from Proposition 4.3 (c) select $r:=\frac{\alpha _0}{(1+\epsilon )\Lambda }$ and $W_r^\varphi (y_k)$ so that $\mathring{W}_r^\varphi (y_k)\cap F_u =\emptyset$ and $x_k\in \Gamma _u \cap \partial W_r^\varphi (y_k).$ Since $\nu _{E_u}(x_k)$ is an “outer normal” also to $W_r^\varphi (y_k)$ at $x_k$ and $W^\varphi$ has no vertical facets, it follows that

$$ \left\langle x_k-y_k,\, \textbf{e}_2\right\rangle \rightarrow 0. $$

In particular,

4.15 $$\begin{aligned} x_k-y_k = s_k \textbf{e}_1 + t_k\textbf{e}_2 \quad \text {with } s_k\rightarrow -\tfrac{r}{\varphi (\textbf{e}_1)} \text { and } t_k\rightarrow 0. \end{aligned}$$

First assume that, up to a subsequence, not relabeled, $y_k\in (-a,a]\times \mathbb {R}$ for all k≥1, see Fig. 2a. Then, recalling that $\mathring{W}_r^\varphi (y_k)\cap (I\times \mathbb {R})\subset E_u,$ we have

4.16 $$\begin{aligned} \Vert u\Vert _\infty \ge \left\langle y_k + \tfrac{r\textbf{e}_2}{\varphi (\textbf{e}_2)},\, \textbf{e}_2\right\rangle = \left\langle y_k,\, \textbf{e}_2\right\rangle + \tfrac{r}{\varphi (\textbf{e}_2)} = \left\langle x_k,\, \textbf{e}_2\right\rangle + \tfrac{r}{\varphi (\textbf{e}_2)}-t_k, \end{aligned}$$

where $y_k + \tfrac{r\textbf{e}_2}{\varphi (\textbf{e}_2)}$ is the top point of $W_r^\varphi (y_k)$ in the vertical direction and in the last equality we used (4.15). As $x_k$ lies on the graph of *u*,  $\left\langle x_k,\, \textbf{e}_2\right\rangle \ge -\Vert u\Vert _\infty ,$ and thus, from (4.16) and the definition of *r* we deduce

$$ 2\Vert u\Vert _\infty \ge \tfrac{\alpha _0}{(1+\epsilon )\Lambda \varphi (\textbf{e}_2)}-t_k. $$

Now first letting $k\rightarrow +\infty$ and then $\epsilon \rightarrow 0^+$ we deduce

$$ \Vert u\Vert _\infty \ge \tfrac{\alpha _0}{2\Lambda \varphi (\textbf{e}_2)}, $$

which contradicts (4.1).

Now assume that $y_k\in (a,+\infty )\times \mathbb {R}$ for all k≥1, see Fig. 2b. In this case, by the partial monotonicity of φ, we have $r>\rho :=a\varphi (\textbf{e}_1)$ and hence $\rho <\frac{\alpha _0}{\Lambda }.$ Applying Proposition 4.3 (c) with r=ρ we can find $W_\rho ^\varphi (y_k')$ such that $\mathring{W}_\rho ^\varphi (y_k')\cap F_u=\emptyset$ with $x_k\in \Gamma _u\cap \partial W_\rho ^\varphi (y_k').$ As above, $\left\langle x_k-y_k',\, \textbf{e}_2\right\rangle \rightarrow 0,$ i.e.,

$$\begin{aligned} x_k-y_k' = s_k' \textbf{e}_1 + t_k'\textbf{e}_2 \quad \text {with } s_k'\rightarrow -\tfrac{\rho }{\varphi (\textbf{e}_1)} \text { and } t_k'\rightarrow 0. \end{aligned}$$

Since $\mathring{W}_\rho ^\varphi (y_k')\cap (I\times \mathbb {R})\subset E_u,$ we can repeat the same arguments leading to (4.16) to get

$$ \Vert u\Vert _\infty \ge \left\langle y_k' + \tfrac{\rho \textbf{e}_2}{\varphi (\textbf{e}_2)},\, \textbf{e}_2\right\rangle = \left\langle y_k',\, \textbf{e}_2\right\rangle + \tfrac{\rho }{\varphi (\textbf{e}_2)} = \left\langle x_k,\, \textbf{e}_2\right\rangle + \tfrac{\rho }{\varphi (\textbf{e}_2)}-t_k' \ge -\Vert u\Vert _\infty + \tfrac{\rho }{\varphi (\textbf{e}_2)}-t_k'. $$

Now letting $k\rightarrow +\infty$ and recalling the definition of ρ we get

$$ \Vert u\Vert _\infty \ge \tfrac{a\varphi (\textbf{e}_1)}{2\Lambda \varphi (\textbf{e}_2)} = \tfrac{|I|\varphi (\textbf{e}_1)}{4\Lambda \varphi (\textbf{e}_2)}, $$

which again contradicts (4.1).

These contradictions show the validity of (4.14). In view of (4.14) and [19, Lemma 3.10], it follows that $\Gamma _u:=(I\times \mathbb {R})\cap \partial E_u$ is the graph of a Lipschitz function (with a Lipschitz constant $\sqrt{1/\lambda ^2-1}$) in the vertical direction. Thus, *u* admits a Lipschitz representative. □

Now we are ready to prove the regularity of Λ-local minimizers.

### Proof of Theorem 4.1

By Corollary 4.4, *u* is Lipschitz continuous in *I*. Assume now φ is $$C^2,$$ elliptic and partially monotone. Then so is $$\varphi ^o.$$ Moreover, the boundary of $$W^\varphi $$ is a closed $$C^2$$-curve without segments. By Proposition 4.3, the subgraph $$E_u:=\textrm{sg}(u)$$ of *u* satisfies uniform^4^ interior and exterior φ-ball conditions at every point of $$(I\times \mathbb {R}) \cap \partial E_u.$$ In view of Proposition 2.2 (c) this implies that $$E_u$$ and $$F_u$$ satisfy the classical ball condition of radius ρ>0 with a suitable ρ>0 depending only on $$\alpha _0,$$ Λ and the constant $$\bar{r}$$ in Proposition 2.2 (c). This allows us to obtain an $$L^\infty $$-bound for the second derivative of *u* in terms of 1/ρ, which yields $u^{\prime }$ is also Lipschitz in *I*,  see for instance [12, Sect. 2] for details. This and the Lipschitz continuity of *u* imply $$u\in C^{1,1}(I).$$ □

### Some generalizations

In this section we relax the Λ-local minimality assumption on *u* in Theorem 4.1. To this aim, we start with the following

#### Definition 4.5

*(*$(\gamma ,\Lambda )$*-local minimizer)* Given an anisotropy φ on $\mathbb {R}^2,$ γ>0, $\Lambda \ge 0,$ and a bounded open interval $I\subset \mathbb {R},$ we say a function $u\in BV_{\textrm{loc}}(I)\cap L^\infty (I)$ is a $(\gamma ,\Lambda )$*-local minimizer* provided that its subgraph $E_u:=\textrm{sg}(u)$ satisfies

4.17 $$\begin{aligned} P_\varphi (E_u,\Omega ) \le P_\varphi (F,\Omega ) + \Lambda |E_u\Delta F| \end{aligned}$$

for any open set $\Omega \Subset I\times (-\gamma ,\gamma )$ and $F\in BV_{\textrm{loc}}(I\times \mathbb {R};\{0,1\})$ with $E_u\Delta F\Subset \Omega .$

Notice that $(\gamma ,\Lambda )$-local minimizers are not necessarily Λ-local minimizers, as local perturbations are taken only in $I\times (-\gamma ,\gamma ).$ Still, we can readily check that the density estimates in Lemma 2.9 and properties in (2.7) hold, and therefore we can speak about closed and open representatives of $E_u.$ Moreover, in case *I* and *u* are bounded, we can apply (4.17) with the set *D* in the proof of Proposition 4.3 provided for instance

4.18 $$\begin{aligned} \gamma > \frac{\alpha _0\varphi (\textbf{e}_1)}{2\Lambda }; \end{aligned}$$

for such γ, if φ is partially monotone and *u* satisfies (4.10), all assertions of Proposition 4.3 are valid. This was sufficient to prove Theorem 4.1. Thus, we have shown:

#### Theorem 4.6

Let φ be a partially monotone anisotropy on $\mathbb {R}^2$ such that $W^\varphi$ does not have vertical facets, γ>0 satisfy (4.18), Λ>0 and $I\subset \mathbb {R}$ be a bounded interval. Then every $(\gamma ,\Lambda )$-local minimizer *u* of $\mathscr {A}_\varphi$ in *I*,  satisfying the $L^\infty$-bound (4.1), is Lipschitz continuous in *I*. If, additionally, φ is elliptic and $C^2,$ then $u\in C^{1,1}(I).$

## Applications

### Minimizers of the perturbed area

Let φ be an anisotropy on $\mathbb {R}^2,$ $I \subset \mathbb {R}$ be a bounded open interval and $g\in L^\infty (I).$ Given p≥1, consider the functional in (1.7), i.e.,

$$ \mathscr {G}(u):=\mathscr {A}_\varphi (u,I) + \int _I|u-g|^pds,\quad u\in L^1(I), $$

where we set $\mathscr {G}(u)=+\infty$ if u∉BV(I).

#### Lemma 5.1

There exists a minimizer $u\in L^1(I)$ of $\mathscr {G}.$ Moreover, u∈BV(I) and $\Vert u\Vert _\infty \le \Vert g\Vert _\infty .$ Finally, if p>1, then *u* is unique.

If p=1, then in general minimizers are not unique, see the Introduction.

#### Proof

The proof is standard and we provide it for completeness. Let $(u_k) \subset L^1(I)$ be a minimizing sequence. We may assume $\mathscr {G}(u_k) \le \mathscr {G}(0)$ for all *k*,  therefore,

$$ \mathscr {A}_\varphi (u_k,I)\le \mathscr {G}(0)\quad \text {and}\quad \int _I | u_k -g|^pds\le \mathscr {G}(0). $$

By the convexity of $\varphi ^o$ and (2.1) we have

$$ \mathscr {G}(0)\ge \mathscr {A}_\varphi (u_k,I) \ge \int _I \varphi ^o(Du_k) - \varphi ^o(\textbf{e}_2)|I| \ge c \int _I |Du_k| - \varphi ^o(\textbf{e}_2)|I|. $$

Moreover, by the Hölder inequality,

$$\begin{aligned} \int _I|u_k|ds  &   \le \int _I|u_k-g|ds+\Vert g\Vert _\infty |I| \le \Big (\int _I|u_k-g|^pds\Big )^{1/p} |I|^{1-\frac{1}{p}} + \Vert g\Vert _\infty |I|\\  &   \le (\mathscr {G}(0))^{1/p} \vert I\vert ^{1-\frac{1}{p}} +\Vert g\Vert _\infty |I|. \end{aligned}$$

Thus, the sequence $(u_k)_k$ is bounded in *BV*(*I*) and by the $L^1$-compactness in *BV*,  up to a subsequence, not relabeled, $u_k\rightarrow u$ in $L^1(I)$ for some u∈BV(I). By the Riesz-Fischer lemma, we may also assume $u_k\rightarrow u$ a.e. in *I*. Then by the $L_{\textrm{loc}}^1(I)$-lower semicontinuity of $\mathscr {A}_\varphi (\cdot ,I),$

$$ \liminf _{k\rightarrow +\infty } \mathscr {A}_\varphi (u_k,I) \ge \mathscr {A}_\varphi (u,I) $$

and by the Fatou’s lemma

$$ \liminf _{k\rightarrow +\infty } \int _I|u_k-g|^pds\ge \int _I|u-g|^pds. $$

Therefore, u∈BV(I) is a minimizer of $\mathscr {G}.$ In particular, $u \in L^\infty (I)$.

To show that $\Vert u\Vert _\infty \le \Vert g\Vert _\infty ,$ let $v:=\max \{u,-\Vert g\Vert _\infty \}.$ Since |u-g|≥|v-g| a.e. in *I*,  we have

$$ \int _I|u-g|^pds\ge \int _I|v-g|^pds$$

with the strict inequality if the set $\{u<-\Vert g\Vert _\infty \}$ has positive measure. Moreover, by Lemma 2.4

$$ \int _I\varphi ^o(-Du,1) = P_\varphi (\textrm{sg}(u),I\times \mathbb {R}) =P_{\varphi }(\textrm{epi}(u),I\times \mathbb {R}). $$

Observe that

$$ P_{\varphi }(\textrm{epi}(v),I\times \mathbb {R}) = P_{\varphi }(\textrm{epi}(u) \cap [I\times (-\Vert g\Vert _\infty ,+\infty )],I\times \mathbb {R}) \le P_{\varphi }(\textrm{epi}(u),I\times \mathbb {R}), $$

where in the last inequality we used a cutting with half-spaces argument, see e.g. [4]. Hence

$$ \mathscr {A}_\varphi (u,I) = P_{\varphi }(\textrm{epi}(u),I\times \mathbb {R}) \ge P_{\varphi }(\textrm{epi}(v),I\times \mathbb {R}) \ge \mathscr {A}_\varphi (v,I). $$

Thus $\mathscr {G}(u)\ge \mathscr {G}(v)$ with the strict inequality if the set $\{u<-\Vert g\Vert _\infty \}$ has positive measure. Then the minimality of *u* implies $u\ge -\Vert g\Vert _\infty$ a.e. in *I*. Similarly, we can show $u\le \Vert g\Vert _\infty$ a.e. in *I*.

Let us check the uniqueness of minimizers when p>1. Consider any minimizers u,v∈BV(I) and $\alpha \in (0,1).$ We have

$$\begin{aligned}&\mathscr {A}_\varphi (\alpha u + (1-\alpha )v, I)\\&= \sup _{h=(h_1,h_2) \in C_c^1(I; W^\varphi )} \Big (\alpha \int _I (uh_1' + h_2) ds+ (1 - \alpha )\int _I (vh_1' + h_2)ds\Big ) \\&\le \alpha \sup _{h=(h_1,h_2) \in C_c^1(I; W^\varphi )} \int _I (uh_1' + h_2)ds+ (1 - \alpha ) \sup _{h=(h_1,h_2) \in C_c^1(I; W^\varphi )} \int _I (vh_1' + h_2)ds\\&= \alpha \mathscr {A}_\varphi (u, I) + (1 - \alpha ) \mathscr {A}_\varphi (v, I). \end{aligned}$$

Therefore, $\mathscr {A}_\varphi$ is convex. Since p>1, the $L^p$-norm is strictly convex, and hence, in case $\alpha \in (0,1)$ and $\{u\ne v\}$ has positive measure, we obtain

$$ \mathscr {G}(\alpha u + (1-\alpha )v) < \alpha \mathscr {G}(u) + (1-\alpha ) \mathscr {G}(v) = \min \, \mathscr {G}, $$

a contradiction. This shows u=v a.e. on *I*,  i.e., the minimizer is unique. □

#### Remark 5.2

In fact,

$$ -\Vert g^-\Vert _\infty \le -\Vert u^-\Vert _\infty \le \Vert u^+\Vert _\infty \le \Vert g^+\Vert _\infty , $$

where $a^\pm =\max \{\pm a,0\}$ are the positive and negative parts of *a*.

The next proposition establishes a bridge between minimizers of $\mathscr {G}$ and $(\gamma ,\Lambda )$-minimizers of $\mathscr {A}_\varphi .$

#### Proposition 5.3

Assume that φ is partially monotone and let u∈BV(I) be a minimizer of $\mathscr {G}.$ Then *u* is a $(\gamma ,\Lambda )$-minimizer of $\mathscr {A}_\varphi$ in *I* for any $\gamma >2\Vert g\Vert _\infty ,$ where

$$ \Lambda :=p(\gamma + \Vert g\Vert _\infty )^{p-1}. $$

#### Proof

By Lemma 5.1, $\Vert u\Vert _\infty \le \Vert g\Vert _\infty .$ Let $E_u:=\textrm{sg}(u)$ be the subgraph of *u* and consider any $F\in BV_{\textrm{loc}}(I\times \mathbb {R};\{0,1\})$ with $E_u\Delta F\Subset I\times (-\gamma ,\gamma ).$ Let *v* be the vertical rearrangement of *F* as in the proof of Proposition 2.7. By construction, $v\in BV_{\textrm{loc}}(I),$ $\mathrm {supp\,}(u-v)\Subset I$ and hence v∈BV(I). In addition, by the Fubini-Tonelli’s theorem and the partial monotonicity of φ,

5.1 $$\begin{aligned} |E_u\Delta F| = \int _I |u-v|ds\quad \text {and}\quad P_\varphi (F,I\times \mathbb {R}) \ge P_\varphi (\textrm{sg}(v),I\times \mathbb {R}) = \mathscr {A}_\varphi (v,I), \nonumber \\ \end{aligned}$$

again see for instance the proof of Proposition 2.7. Moreover, since $\gamma >2\Vert u\Vert _\infty$ and *F* does not cross the horizontal sides of the rectangle $I\times (-\gamma ,\gamma ),$ we have $\Vert v\Vert _\infty < \gamma .$ Thus, by the minimality of *u* and (5.1),

5.2 $$\begin{aligned}  &   P_\varphi (E_u,I\times \mathbb {R}) = \mathscr {A}_\varphi (u,I) \le \mathscr {A}_\varphi (v,I) + \int _I\Big (|v-g|^p -|u-g|^p\Big )ds\nonumber \\  &   \quad \le P_\varphi (F,I\times \mathbb {R}) + p\max \{|v-g|^{p-1},|u-g|^{p-1}\} \int _I|u-v|ds\le P_\varphi (F,I\times \mathbb {R}) + \Lambda |E_u\Delta F|, \end{aligned}$$

where in the last inequality we used

$$\begin{aligned} \max \{|v-g|^{p-1},|u-g|^{p-1}\}\le&\max \{(\Vert v\Vert _\infty + \Vert g\Vert _\infty )^{p-1},(\Vert u\Vert _\infty + \Vert g\Vert _\infty )^{p-1}\} \\ \le&\max \{(\gamma + \Vert g\Vert _\infty )^{p-1},(2\Vert g\Vert _\infty )^{p-1}\} \le (\gamma + \Vert g\Vert _\infty )^{p-1}. \end{aligned}$$

Since $E_u\Delta F\Subset I\times (-\gamma ,\gamma ),$ comparing (5.2) with (4.17) we conclude that *u* is a $(\gamma ,\Lambda )$-local minimizer of $\mathscr {A}_\varphi .$ □

From Proposition 5.3 and Theorem 4.6 we deduce the following

#### Theorem 5.4

(Regularity of minimizers) Let φ be a partially monotone anisotropy on $\mathbb {R}^2$ such that $W^\varphi$ does not have vertical facets, $I\subset \mathbb {R}$ be a bounded open interval and p≥1. Let

5.3 $$\begin{aligned} \sigma : = \Big ( \tfrac{1}{4^{p-1}p}\,\min \Big \{\tfrac{\alpha _0 \varphi (\textbf{e}_1)}{4},\, \tfrac{\alpha _0}{2\varphi (\textbf{e}_2)},\, \tfrac{|I|\varphi (\textbf{e}_1)}{4\varphi (\textbf{e}_2)}\Big \}\Big )^{1/p}, \end{aligned}$$

where $\alpha _0>0$ is defined in (4.2). Let $g\in L^\infty (I)$ be such that

5.4 $$\begin{aligned} \Vert g\Vert _\infty <\sigma . \end{aligned}$$

Then every minimizer *u* of the functional $\mathscr {G}$ in (1.7) is Lipschitz continuous in *I*. Moreover, if φ is elliptic and $C^2,$ then $u\in C^{1,1}(I).$

#### Proof

Let $\gamma :=3\sigma$ and $\Lambda :=(\gamma + \sigma )^{p-1}p = (4\sigma )^{p-1}p>0.$ By (5.3),

$$ \sigma = \min \Big \{\tfrac{\alpha _0\varphi (\textbf{e}_1)}{4\Lambda },\,\tfrac{\alpha _0}{2\Lambda \varphi (\textbf{e}_2)},\,\tfrac{|I|\varphi (\textbf{e}_1)}{4\Lambda \varphi (\textbf{e}_2)}\Big \}. $$

Thus, γ satisfies (4.18). Since $\Vert g\Vert _\infty < \sigma =\gamma /3,$ by Proposition 5.3*u* is a $(\gamma ,\Lambda )$-local minimizer of $\mathscr {A}_\varphi$ in *I*. Moreover, by Lemma 5.1, $\Vert u\Vert _\infty \le \Vert g\Vert _\infty$ and hence, from (5.3) and (5.4) it follows that *u* satisfies (4.1). Now the assertions directly follow from Theorem 4.6. □

When φ is Euclidean and p=1, (5.3) reads as

5.5 $$\begin{aligned} \sigma = \frac{1}{4}\,\min \Big \{ \alpha _0, |I|\Big \}, \end{aligned}$$

where $\alpha _0=\frac{2\sqrt{\pi }}{4\pi +1}.$ Thus, Theorem 5.4 implies that every minimizer of $\mathscr {G}$ belongs to $C^{1,1}(I)$ provided that $\Vert g\Vert _\infty < \sigma .$ This gives a positive answer to Conjecture 1.1 in case n=k=1, except for the dependence of σ on |*I*|. Note that our σ depends on |*I*|,  while the σ of [5] (with p=2) depends only on $1/\sqrt{|I|}.$

The following example shows that in general the local $C^{1,1}$-regularity of *u* in Theorem 5.4 cannot be improved.

#### Example 5.5

**Fig. 3:**
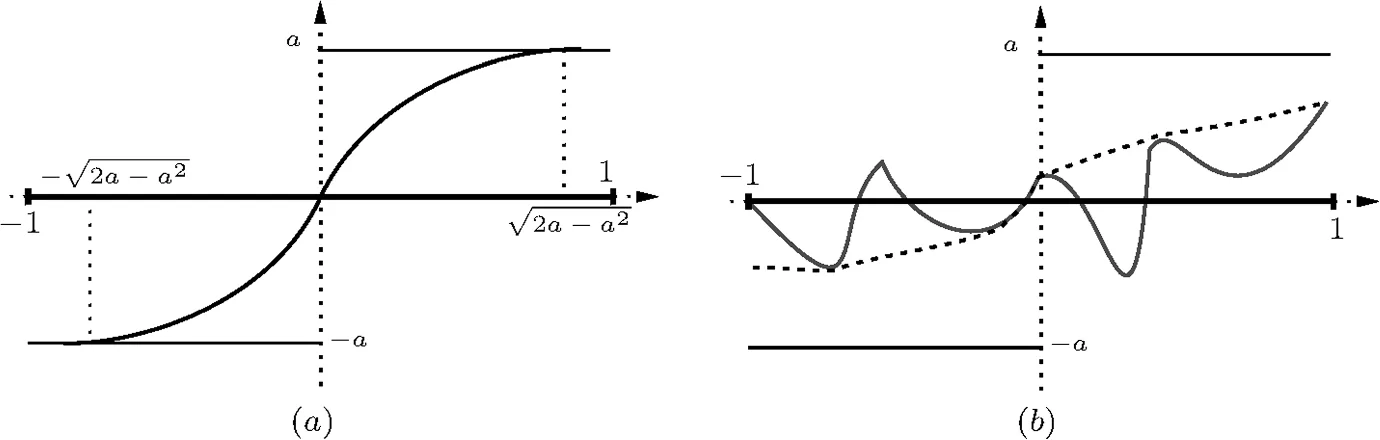
The graph of *u* in (**a**) and convex/concave nondecreasing envelope of *v* in (**b**)

Let I=(-1,1), φ be Euclidean, p=1 and

$$ g(s)= {\left\{ \begin{array}{ll} a &  \text {if } s\in (0,1),\\ -a &  \text {if } s\in (-1,0) \end{array}\right. } $$

for some a∈(0,1). Then the $C^{1,1}(I)\setminus C^2(I)$-function (see Fig. 3a)

$$ u(s)= {\left\{ \begin{array}{ll} a &  \text {if } s\in [\sqrt{2a-a^2},1), \\ a-1+\sqrt{1-(s-\sqrt{2a-a^2})^2} \quad &  \text {if } s\in [0,\sqrt{2a-a^2}],\\ 1-a-\sqrt{1-(s+\sqrt{2a-a^2})^2} &  \text {if } s\in [-\sqrt{2a-a^2},0],\\ -a &  \text {if } s\in (-1,-\sqrt{2a-a^2}] \end{array}\right. } $$

is the unique minimizer of $\mathscr {G}.$ Indeed, for simplicity, set

$$ h(s):=\frac{u'(s)}{\sqrt{1+{u'(s)}^2}} = {\left\{ \begin{array}{ll} 0 &  \text {if } s\in [\sqrt{2a-a^2},1]\\ -s+\sqrt{2a-a^2} \quad &  \text {if } s\in [0,\sqrt{2a-a^2}]\\ s+\sqrt{2a-a^2} &  \text {if } s\in [-\sqrt{2a-a^2},0]\\ 0 &  \text {if } s\in [-1,-\sqrt{2a-a^2}] \end{array}\right. } $$

so that $h\in {\textrm{Lip}}([-1,1])\cap C^\infty ([-1,1]\setminus \{0,\pm \sqrt{2a-a^2}\})$ with

5.6 $$\begin{aligned} h'= {\left\{ \begin{array}{ll} 0 &  \text {a.e. in } \{u=g\},\\ -1 &  \text {a.e. in } \{u<g\},\\ 1 &  \text {a.e. in } \{u>g\}. \end{array}\right. } \end{aligned}$$

As h(±1)=0, for any v∈BV(-1,1), integrating by parts we have

$$\begin{aligned}  &   \int _{-1}^1 (u-v)h'ds= (u-v)h\big |_{-1}^1 - \int _{-1}^1 hu'ds+\int _{-1}^1\,h\,dDv \\  &   \quad =-\int _{-1}^1 \frac{{u'}^2ds}{\sqrt{1+{u'}^2}} + \int _{-1}^1 \frac{u'dDv}{\sqrt{1+{u'}^2}} = -\int _{-1}^1 \sqrt{1+{u'}^2}ds+ \int _{-1}^1 \frac{ds+ u'dDv}{\sqrt{1+{u'}^2}}. \end{aligned}$$

On the other hand, by the explicit expression of $h^{\prime },$

$$ \int _{-1}^1 (u-v)h'ds=\int _{-1}^1 (u-g)h'ds+\int _{-1}^1 (g-v)h'ds=\int _{-1}^1 |u-g|ds+ \int _{-1}^1(g-v)h'ds. $$

Combining these two equalities, we deduce

5.7 $$\begin{aligned} \int _{-1}^1 \sqrt{1+{u'}^2}ds+ \int _{-1}^1 |u-g|ds= \int _{-1}^1 \frac{ds+u'dDv}{\sqrt{1+{u'}^2}}+\int _{-1}^1(v-g)h'ds. \end{aligned}$$

By the Hölder inequality^5^ and the bound $\Vert h'\Vert _\infty \le 1,$

$$ \int _{-1}^1 \frac{ds+u'dDv}{\sqrt{1+{u'}^2}} \le \int _{-1}^1\sqrt{1+|Dv|^2}\quad \text {and}\quad \int _{-1}^1(v-g)h'ds\le \int _{-1}^1|v-g|ds. $$

Therefore, from (5.7) we deduce $\mathscr {G}(u) \le \mathscr {G}(v).$

Next, let us show the uniqueness of *u*.

Let v∈BV(-1,1) be any other minimizer of $\mathscr {G}.$ By Remark 5.2, $-a\le v\le a.$ Let $v^*:[0,1]\rightarrow \mathbb {R}$ be the smallest nondecreasing concave function with $v^*\ge v$ a.e. in [0, 1] and $v_*$ be the largest nondecreasing convex function with $v_*\le v$ a.e. in [0, 1]. Set $\bar{v}:=v^*\chi _{(0,1]}+v_*\chi _{[-1,0)}.$ As $g-v \ge g-\bar{v}\ge 0$ in (0, 1) and $v-g \ge \bar{v}-g\ge 0$ in (-1,0), we have

$$ \int _{-1}^1 |g-v|ds\ge \int _{-1}^1 |g-\bar{v}|ds$$

with strict inequality if $\{v\ne \bar{v}\}$ has positive Lebesgue measure. Moreover, as we are replacing nonconcave/nonconvex parts of the graph of *v* with line segments (see Fig. 3b), $\mathscr {A}_\varphi (v,I) \ge \mathscr {A}_\varphi (\bar{v},I).$ Thus, $\mathscr {G}(\bar{v})\le \mathscr {G}(v).$ This inequality shows that we may assume $v=\bar{v}.$ In this case, by (2.2)

$$ \int _{-1}^1 \frac{u'\,dDv}{\sqrt{1+{u'}^2}} = \int _{-1}^1\frac{u'v'ds}{\sqrt{1+{u'}^2}} + \sqrt{2a-a^2}\, (v^+(0)-v^-(0)). $$

Thus, as above, from (5.7), the Hölder inequality and (5.6) we get

5.8 $$\begin{aligned} \mathscr {G}(u)&= \int _{-1}^1 \sqrt{1+{u'}^2}ds+ \int _{-1}^1 |u-g|ds\nonumber \\&= \int _{-1}^1 \frac{(1+u'v')ds}{\sqrt{1+{u'}^2}}+ \sqrt{2a-a^2}\, (v^+(0)-v^-(0)) +\int _{-1}^1|v-g|ds\nonumber \\&\le \int _{-1}^1 \sqrt{1+{v'}^2}ds+ \sqrt{2a-a^2}\,(v^+(0)-v^-(0)) +\int _{-1}^1|v-g|ds\le \mathscr {G}(v), \end{aligned}$$

where in the last inequality we used $$\sqrt{2a-a^2}<1.$$ Since both *u* and *v* are minimizers, all inequalities in (5.8) are in fact equalities. In particular, $u^{\prime }=v^{\prime }$ a.e. in (-1,1) and $$v^+(0)=v^-(0).$$ This implies u=v+C for some real constant *C*. Then, recalling $$u(\pm 1)=\pm a$$ and $$-a\le v\le a,$$ we deduce C=0, i.e., u=v.

Notice that Example 5.5 shows that the threshold σ in (5.5) is not optimal, in general.

## Data Availability

The paper has no associated data.

## Footnotes

1 In fact, ω-minimal sets are defined for any anisotropy, not necessarily even and [20, Theorem 3.4] shows that in general Σ is discrete. Moreover, if $W^\varphi$ is neither a triangle nor a quadrilateral, then Σ is empty.

2 I.e., their normal belongs to $\partial \varphi (\textbf{e}_1).$

3 Possibly out of a discrete set when $W^\varphi$ is a quadrilateral.

4 The radii of the tangent Wulff shapes can be chosen a constant $r\in (0,\frac{\alpha _0}{\Lambda })$ along the graph of *u*.

5 If λ and μ are bounded Radon measures in a bounded open set $I\subset \mathbb {R}$ and $p,q\in C(\overline{I}),$ there holds

$$ \int _I pd\mu + qd\lambda \le \int _I\sqrt{p^2+q^2}\, d\sqrt{\mu ^2+\lambda ^2}, $$

where

$$ \int _J \sqrt{\mu ^2+\lambda ^2}:=\sup \Big \{\int _J \phi d\mu + \psi d\lambda :\,\, (\phi ,\psi )\in C_c(J;\mathbb {R}^2),\,\, \Vert \phi ^2+\psi ^2\Vert _\infty \le 1\Big \} $$

is the total variation of $(\mu ,\lambda )$ in the open set J⊆I. We apply this inequality with $p=(1+{u'}^2)^{-1/2},$ $q=u'(1+{u'}^2)^{-1/2},$ $\mu =\mathcal {L}^1$ and λ=Dv.
